# Selective induction of Rab9-dependent alternative mitophagy using a synthetic derivative of isoquinoline alleviates mitochondrial dysfunction and cognitive deficits in Alzheimer's disease models

**DOI:** 10.7150/thno.88718

**Published:** 2024-01-01

**Authors:** Jee-Hyun Um, Dong Jin Shin, Se Myeong Choi, Alen Benhur Pravin Nathan, Young Yeon Kim, Da Ye Lee, Dae Jin Jeong, Dong Hyun Kim, Kyung Hwa Kim, Young Hye Kim, Jihoon Nah, Jeong-hee Jeong, Eunhee Yoo, Hwa Kyoung Shin, Hwan Tae Park, Jihoon Jo, Jong Hyun Cho, Jeanho Yun

**Affiliations:** 1 Peripheral Neuropathy Research Center, College of Medicine, Dong-A University, Busan, Republic of Korea.; 2 Department of Biochemistry, College of Medicine, Dong-A University, Busan, Republic of Korea.; 3 Department of Translational Biomedical Sciences, Graduate School of Dong-A University, Busan, Republic of Korea.; 4 Department of Medicinal Biotechnology, College of Health Sciences, Dong-A University, Busan, Republic of Korea.; 5 Department of Biomedical Sciences, Chonnam National University Medical School, Gwangju, Republic of Korea.; 6 Department of Pharmacology and Department of Advanced Translational Medicine, School of Medicine, Konkuk University, Seoul, Republic of Korea.; 7 Department of Health Sciences, The Graduate School of Dong-A University, 840 Hadan-dong, Saha-gu, Busan 49315, Republic of Korea.; 8 Biomedical Omics Group, Korea Basic Science Institute, Cheongju, Chungbuk, 28119, Republic of Korea.; 9 Department of Biochemistry, Chungbuk National University, Cheongju, Republic of Korea.; 10 Altmedical Co., Ltd. Seoul, 02792, Republic of Korea.; 11 Department of Korean Medical Science, School of Korean Medicine, Pusan National University, Yangsan, Republic of Korea.; 12 Department of Molecular Neuroscience, College of Medicine, Dong-A University, Busan, Republic of Korea.

**Keywords:** mitophagy inducer, alternative mitophagy, Alzheimer's disease, mitochondrial dysfunction, Rab9

## Abstract

**Rationale:** Promotion of mitophagy is considered a promising strategy for the treatment of neurodegenerative diseases including Alzheimer's disease (AD). The development of mitophagy-specific inducers with low toxicity and defined molecular mechanisms is essential for the clinical application of mitophagy-based therapy. The aim of this study was to investigate the potential of a novel small-molecule mitophagy inducer, ALT001, as a treatment for AD.

**Methods:** ALT001 was developed through chemical optimization of an isoquinolium scaffold, which was identified from a chemical library screening using a mitophagy reporter system. *In vitro* and *in vivo* experiments were conducted to evaluate the potential of ALT001 as a mitophagy-targeting therapeutic agent and to investigate the molecular mechanisms underlying ALT001-induced mitophagy. The therapeutic effect of ALT001 was assessed in SH-SY5Y cells expressing mutant APP and mouse models of AD (5×FAD and PS2APP) by analyzing mitochondrial dysfunction and cognitive defects.

**Results:** ALT001 specifically induces mitophagy both *in vitro* and *in vivo* but is nontoxic to mitochondria. Interestingly, we found that ALT001 induces mitophagy through the ULK1-Rab9-dependent alternative mitophagy pathway independent of canonical mitophagy pathway regulators such as ATG7 and PINK1. Importantly, ALT001 reverses mitochondrial dysfunction in SH-SY5Y cells expressing mutant APP in a mitophagy-dependent manner. ALT001 induces alternative mitophagy in mice and restores the decreased mitophagy level in a 5×FAD AD model mouse. In addition, ALT001 reverses mitochondrial dysfunction and cognitive defects in the PS2APP and 5×FAD AD mouse models. AAV-mediated silencing of Rab9 in the hippocampus further confirmed that ALT001 exerts its therapeutic effect through alternative mitophagy.

**Conclusion:** Our results highlight the therapeutic potential of ALT001 for AD via alleviation of mitochondrial dysfunction and indicate the usefulness of the ULK1-Rab9 alternative mitophagy pathway as a therapeutic target.

## Introduction

Mitochondrial dysfunction is closely associated with the development and progression of various neurodegenerative diseases, including Alzheimer's disease (AD) 1-3. Although AD pathogenesis is not fully understood, mounting evidence indicates that mitochondrial dysfunction plays a critical role in the development of AD. Various abnormalities, such as mitochondrial morphology changes, dysregulation of mitochondrial transportation, increased mitochondrial DNA mutation, and decreased activity of key mitochondrial enzymes, have been observed in the brain tissues of AD patients 4. Dysregulated mitochondrial metabolism and biogenesis were also observed in the early phase after the clinical onset of AD and in various AD animal models 5-7, suggesting that mitochondrial dysfunction plays a fundamental role in AD development 4. In addition, many events related to mitochondrial dysfunction, including collapse of the mitochondrial membrane potential, increased mitochondrial ROS production, and impaired ATP generation, are dependent on amyloid precursor protein (APP) and amyloid beta (Aβ) peptide in both *in vitro* and *in vivo* AD models 8-11. These results suggest that mitochondrial dysfunction and major pathological factors, such as Aβ processes, tau hyperphosphorylation, and neuroinflammation, may potentiate each other by forming a vicious cycle during AD development and progression, suggesting that mitochondrial dysfunction may be a potential target for AD treatment 2, 12, 13.

Among the various mitochondrial quality control processes, mitophagy plays a central role in maintaining neuronal mitochondrial function through the degradation of damaged or old mitochondria using autophagy core machinery 14. In mammalian cells, the removal of damaged mitochondria is executed mainly by either the PINK1-Parkin pathway or the PINK1-independent receptor mediated pathway 15, 16. In addition, the ULK1-Rab9-dependent 'alternative mitophagy pathway' has been identified as responsible for maintaining mitochondrial function 17, 18, highlighting the complexity of mitophagy regulatory networks for the maintenance of physiological mitochondrial homeostasis 19, 20. Due to its essential role in mitochondrial quality control, the stimulation of mitophagy has emerged as a novel strategy for the treatment of neurodegenerative diseases 14, 21. Rapidly accumulating evidence indicates that impaired mitophagy is common in AD models 13, 22-24. More importantly, decreased mitophagy activity has been observed in AD patients and AD patient-derived iPSCs 25, 26. Recent studies have indeed proven that stimulation of mitophagy using natural and synthetic compounds, including urolithin A, the NAD^+^ precursor nicotinamide mononucleotide (NMN) and nicotinamide riboside (NR), has beneficial effects on mitochondrial dysfunction and even AD-related cognitive defects 25, 27-30.

However, mitophagy-based therapy is limited by the lack of an appropriate mitophagy inducer 14, 31. The development of an effective and nontoxic mitophagy inducer is essential for the clinical application of mitophagy-based therapies for AD 14. The *in vivo* verification of mitophagy induction and identification of the molecular mechanism of mitophagy induction are also critical for the development of mitophagy-based treatments for AD models 14, 31.

Here, through library screening and chemical optimization of a core scaffold structure derived from positive hit compounds, we developed a mitophagy-specific inducer with low mitochondrial and cellular toxicity, ALT001. We revealed that ALT001 induces mitophagy through the ULK1-Rab9-dependent 'alternative mitophagy pathway' independent of the canonical mitophagy pathway. In addition, we showed that activation of mitophagy using ALT001 restored mitophagy levels and alleviated mitochondrial dysfunction and cognitive defects in AD mouse models. Thus, we propose here for the first time that the alternative mitophagy pathway is a promising target for the treatment of neurodegenerative diseases, including AD.

## Results

### The synthetic isoquinoline derivative ALT001 induces mitophagy and subsequent mitochondrial biogenesis

**Figure 1:**
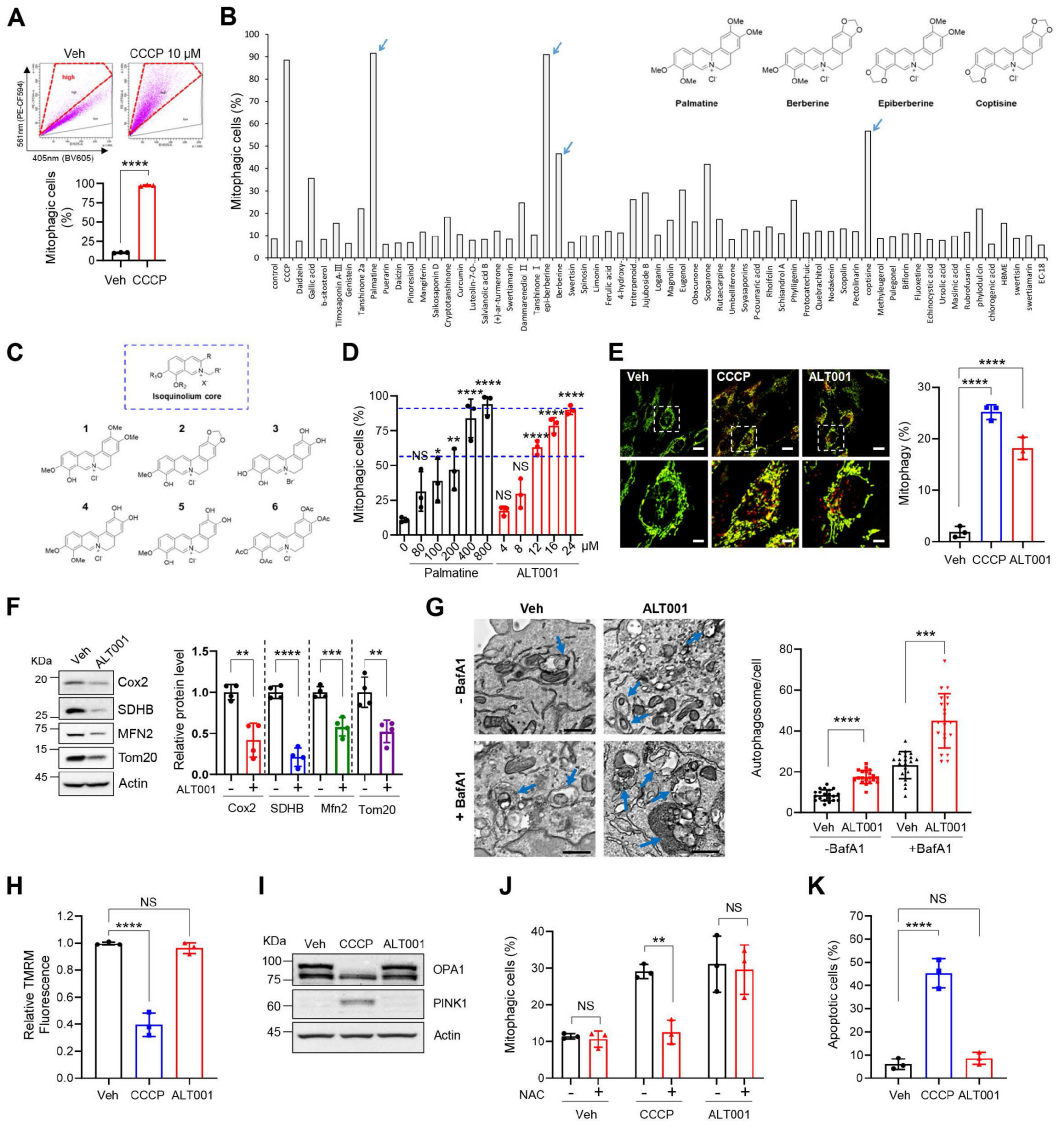
** Development and verification of the mitophagy inducer ALT001. (A)** Mitophagy analysis results using a flow cytometry-based mt-Keima assay in BEAS-2B cells treated with CCCP (10 μM) for 24 h. **(B)** The results of chemical library screening. Arrows indicate the four compounds with an isoquinolium core structure. **(C)** Structure of derivatives with an isoquinolium core scaffold produced through chemical optimization. **(D)** BEAS-2B cells expressing mt-Keima were treated with either palmatine or ALT001 at the indicated concentration for 24 h, and mitophagy levels were analyzed by flow cytometry. **(E-F)** BEAS-2B cells expressing mt-Keima (**E**) or BEAS-2B cells (**F**) were treated with ALT001 (15 μM) or CCCP (10 μM) for 24 h, and mitophagy levels were analyzed by confocal microscopy **(E)**. Scale bars: 20 μm (upper). The boxed regions are enlarged in the bottom panel. Scale bars: 5 μm (bottom). Western blotting analysis was performed using the indicated antibodies **(F)**. **(G)** BEAS-2B cells were treated with ALT001 (15 μM) with or without bafilomycin A1 (BafA1; 100 nM) for 12 h and analyzed by transmission electron microscopy. Scale bars: 1 μm. Arrows indicate autophagosomes containing mitochondria. The autophagosome number per cell is shown on the right as the mean ± SD (n = 20 per group). **(H-I)** BEAS-2B cells were treated with either CCCP (10 μM) or ALT001 (15 μM) for 24 h. The mitochondrial membrane potential was assessed by TMRM staining **(H)**, and western blot analysis was performed using the indicated antibodies **(I)**. **(J)** BEAS-2B cells were treated with either CCCP (10 μM) for 3 h or ALT001 (15 μM) for 9 h, and alone or cotreated with NAC (2 mM) for 3 h. Mitophagy levels were analyzed by flow cytometry.** (K)** BEAS-2B cells were treated with CCCP (10 μM) or ALT001 (15 μM) for 24 h, and apoptotic cells were analyzed by flow cytometry after Annexin V-FITC/PI staining. The results from three biological replicates **(D, E, H, J, K)** or four biological replicates **(F)** are shown as the mean ± SD. Significance was determined by Student's *t test* **(A, F, G)** or one-way **(D, E, H, K)** or two-way ANOVA **(J)** with Šidák's multiple-comparison test. *P < 0.05; **P < 0.01; ***P < 0.001; ****P < 0.0001. NS, not significant.

To identify novel mitophagy inducers that stimulate mitophagy through various potential signaling pathways, we performed chemical screening using the BEAS-2B human nontumorigenic lung epithelial cell line. We adopted a previously established flow cytometric mitophagy assay using pH-dependent mitochondria-targeted Keima (mt-Keima) 32, 33. Because this flow cytometric mitophagy assay can sensitively distinguish cells that are actively undergoing mitophagy, we could conveniently measure the proportion of cells in which mitophagy (mitophagic cells (%)) is activated upon treatment with chemicals, such as carbonyl cyanide m-chlorophenyl hydrazone (CCCP) (**Figure 1A**). To identify nontoxic mitophagy inducers, we screened our in-house chemical library containing 61 widely used single compounds derived from medicinal plants (**Table S1**) in BEAS-2B cells expressing mt-Keima. Interestingly, we noticed that berberine, epiberberine, palmatine, and coptisine containing an isoquinolium core scaffold all exhibited mitophagy-inducing activity (**Figure 1B, Table S1**). To verify the suitability of the isoquinolium core structure for mitophagy inducer development, we modified the parent isoquinolium scaffold by converting phenolic methyl and methylene ethers to the corresponding hydroxyl group, which in turn cumulatively increased the hydrophilicity of the molecules, a powerful and convenient strategy to identify lead compounds for drug discovery (**Figure 1C, Figure S1**). Among these derivatives, one compound generated through the deprotection of all phenolic ether groups on palmatine or berberine had high mitophagy-inducing activity (**Figure S2A**). By comparing this compound with palmatine, we found that mitophagy-inducing activity was improved approximately 25-fold (**Figure 1D**). We named this compound ALT001 and used it for the following study.

Flow cytometric mitophagy assay results demonstrated a significant increase in the percentage of mitophagic cells following ALT001 treatment (**Figure S2B**). In previous studies, we confirmed that the red punctate structure of mt-Keima with a high 561/458-nm ratio corresponds to mitochondria within acidic lysosomal compartments 32, 33. Confocal analysis of mt-Keima fluorescence revealed a significant increase in these red punctate structures upon ALT001 treatment, indicating enhanced mitophagy (**Figure 1E**). Quantitative analysis indicated that ALT001 induced a similar increase in mitophagy compared to CCCP. ALT001-induced mitophagy was further verified by a decrease in the mitoYFP fluorescence signal and mitochondrial proteins and an increase in the number of autophagosomes containing mitochondria (**Figure S2C, Figure 1F-G**). In addition to dose-dependent mitophagy induction (**Figure 1D**), there was a time-dependent increase in the percentage of mitophagic cells (**Figure S2D**). ALT001 also induced mitophagy in cell lines derived from various types of tissues (**Figure S2E-F**). These results indicate that ALT001 efficiently induces mitophagy in human cells.

The clinical application of mitophagy inducers widely used in mitophagy research, such as CCCP, is limited because of their toxicity to mitochondria 34. Thus, we next examined whether ALT001 interferes with mitochondrial function. CCCP treatment (10 μM) dramatically reduced the mitochondrial membrane potential (MMP), but ALT001 did not affect the MMP after up to 24 h of treatment (**Figure 1H**). Cleavage of OPA1, a marker of mitochondrial membrane uncoupling 35, and PINK1 accumulation were also not observed upon ALT001 treatment (**Figure 1I**). Moreover, ALT001-induced mitophagy induction was not inhibited by the antioxidant N-acetyl cysteine (NAC), unlike CCCP-induced mitophagy induction (**Figure 1J**). Cells were exposed to CCCP for a shorter duration (3 h) than ALT001 (9 h) to compare the effect of the antioxidant NAC at a similar mitophagy activity level. In addition, cell death was not induced upon ALT001 treatment, while CCCP markedly induced cell death (**Figure 1K**). These results suggest that ALT001 does not interfere with mitochondrial function or cell viability.

### ALT001 specifically induces mitophagy independent of the canonical mitophagy pathway

**Figure 2:**
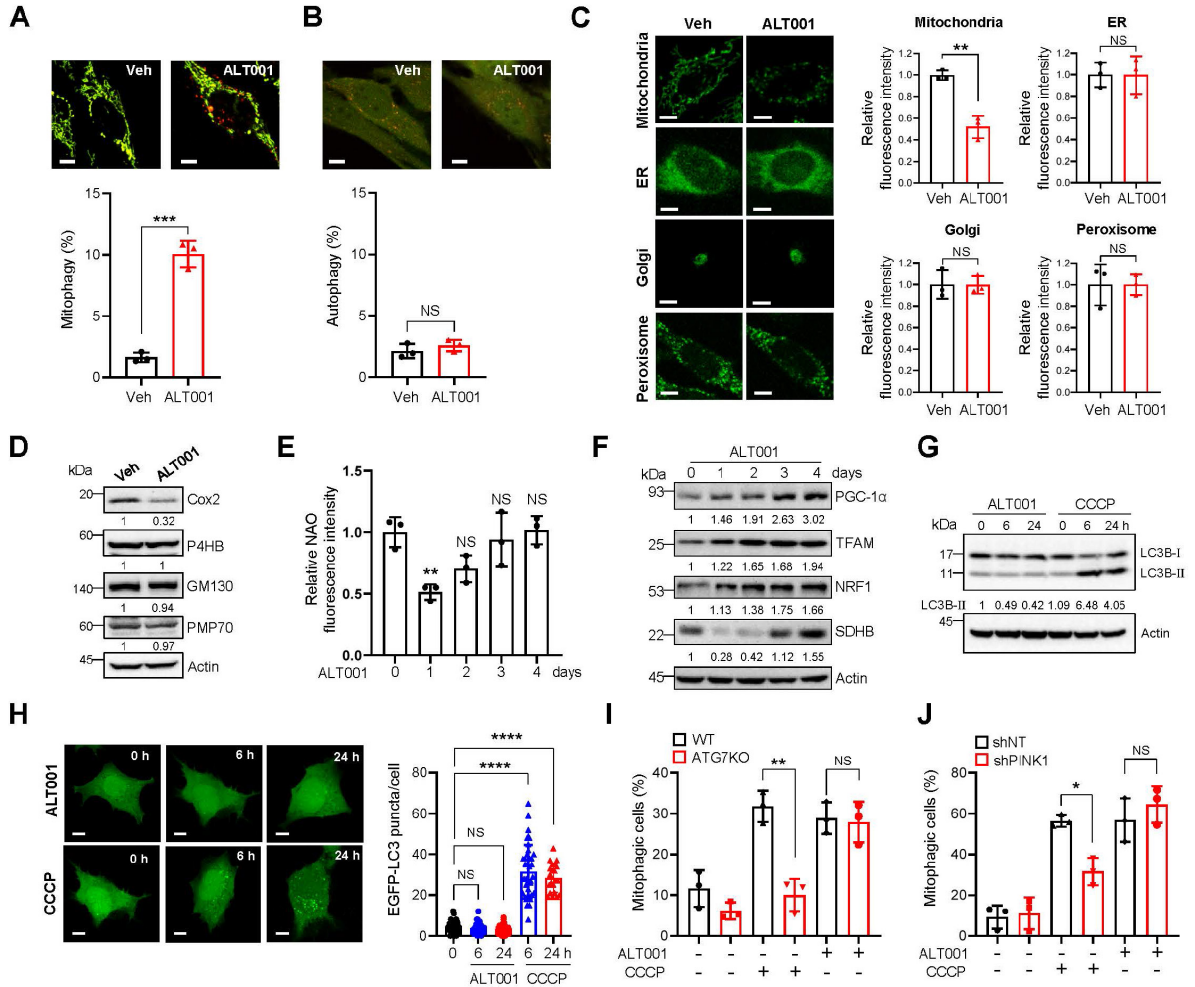
** Specific induction of mitophagy by ALT001 independent of the canonical mitophagy pathway. (A-B)** BEAS-2B cells expressing mt-Keima **(A)** or Keima **(B)** were treated with ALT001 (15 μM) for 18 h, and mitophagy levels were analyzed by confocal microscopy. Scale bar: 10 μm. **(C)** HeLa cells expressing Parkin and mitoYFP, ER-GFP, Golgi-eGFP, or Turquoise2-Peroxi were treated with ALT001 (15 μM) for 24 h. Scale bar: 10 μm. **(D)** BEAS-2B cells were treated with ALT001 (15 μM) for 24 h, and western blot analysis was performed using the indicated antibodies. Numbers below the corresponding blot represent densitometric analysis normalized to Actin. **(E-F)** BEAS-2B cells were treated with ALT001 (15 μM) for 24 h and further incubated until the indicated time points. Mitochondrial mass was measured by NAO staining **(E)**, and western blot analysis was performed using the indicated antibodies **(F)**. Numbers below the corresponding blot represent densitometric analysis normalized to Actin.** (G)** BEAS-2B cells were treated with either CCCP (10 μM) or ALT001 (15 μM) for the indicated time and western blot analysis was performed using LC3B and Actin antibodies. **(H)** HEK293 cells expressing EGFP-LC3 were treated with either CCCP (10 μM) or ALT001 (15 μM) for the indicated time, and EGFP-LC3 puncta were analyzed by confocal microscopy. The LC3 puncta number per cell from three experiments is shown as the mean ± SD (0 h, n = 40; ALT001 6 h, n = 29; 24 h, n = 31; CCCP 6 h, n = 39; CCCP 24 h, n = 21). **(I)** Wild-type (WT) and ATG7 knockout (ATG7 KO) HeLa cells expressing Parkin were treated with either CCCP (10 μM) for 2 h or ALT001 (15 μM) for 24 h, and mitophagy levels were analyzed by flow cytometry. **(J)** BEAS-2B expressing mt-Keima cells expressing control nontargeting shRNA (shNT) or PINK1 shRNA (shPINK1) were treated with either CCCP (10 μM) for 6 h or ALT001 (15 μM) for 24 h, and mitophagy levels were analyzed by flow cytometry. The results from three biological replicates **(A-F, I-J)** or four biological replicates **(G)** are shown as the mean ± SD. Significance was determined by Student's *t test* **(A-C)** or one-way **(E, H)** two-way ANOVA **(I, J)** with Šidák's multiple-comparison test. *P < 0.05; **P < 0.01; ***P < 0.001; ****P < 0.0001. NS, not significant.

To further verify the suitability of ALT001 as a mitophagy-targeting therapeutic agent, we next examined mitophagy-specific induction upon ALT001 treatment. While mitochondria targeted mt-Keima can be used to detect mitophagy induction, the level of macroautophagy can be measured by assessing the characteristic punctate structure formation of Keima protein localized in the cytoplasm 17, 36. We found that ALT001 treatment did not induce the formation of cytosolic Keima puncta, while mt-Keima puncta were readily increased upon ALT001 treatment (**Figure 2A-B**), suggesting that ALT001 does not induce macroautophagy. In addition, analysis of the fluorescent markers for different intracellular organelles upon treatment with ALT001 in HeLa-Parkin cells indicated that only the mitochondrial marker (mito-YFP) signal was decreased, while the ER, Golgi, and peroxisome signals were not changed (**Figure 2C**). Consistently, western blotting showed that the levels of endogenous marker proteins for the ER (P4HB), Golgi (GM130), and peroxisome (PMP70) were not changed upon ALT001 treatment (**Figure 2D, Figure S3A**). These results suggest that ALT001 specifically induces mitophagy.

Mitochondrial content is coordinately regulated by mitophagy and mitochondrial biogenesis 37. Consistently, the results of NAO staining and SDHB protein western blot analysis after 24 h of ALT001 treatment indicated an initial decrease in mitochondrial amounts, followed by a gradual increase (**Figure 2E-F**). Furthermore, critical regulators of mitochondrial biogenesis, such as PGC-1α, TFAM, and NRF1, were induced upon ALT001 treatment (**Figure 2F, Figure S3B**), suggesting that ALT001-induced mitophagy simultaneously stimulates mitochondrial biogenesis.

The PINK1-Parkin pathway mediates canonical mitophagy in various cellular contexts, such as in the presence of CCCP 15, 16. To understand the molecular mechanism of ALT001-induced mitophagy, we first examined the LC3 level upon ALT001 treatment. Interestingly, the level of the LC3B-Ⅱ form, a typical indicator of mitophagy induction, was not changed after ALT001 treatment, whereas a robust increase in the LC3B-Ⅱ form was observed as early as 6 h after CCCP treatment (**Figure 2G, Figure S3C**). In addition, GFP-LC3 puncta formation was not observed after ALT001 treatment (**Figure 2H**). These results suggest that ALT001-induced mitophagy is independent of LC3. Moreover, ALT001-induced mitophagy was not decreased in ATG7 knockout HeLa-Parkin cells, while CCCP-induced mitophagy was abolished (**Figure 2I, Figure S3D**). ALT001-induced mitophagy was also not inhibited by shRNA-mediated knockdown of PINK1, unlike CCCP-induced mitophagy (**Figure 2J, Figure S3E**). These results indicate that ALT001-induced mitophagy is not mediated by the canonical PINK1-Parkin pathway.

### The ULK1-Rab9 pathway mediates ALT001-induced mitophagy

**Figure 3:**
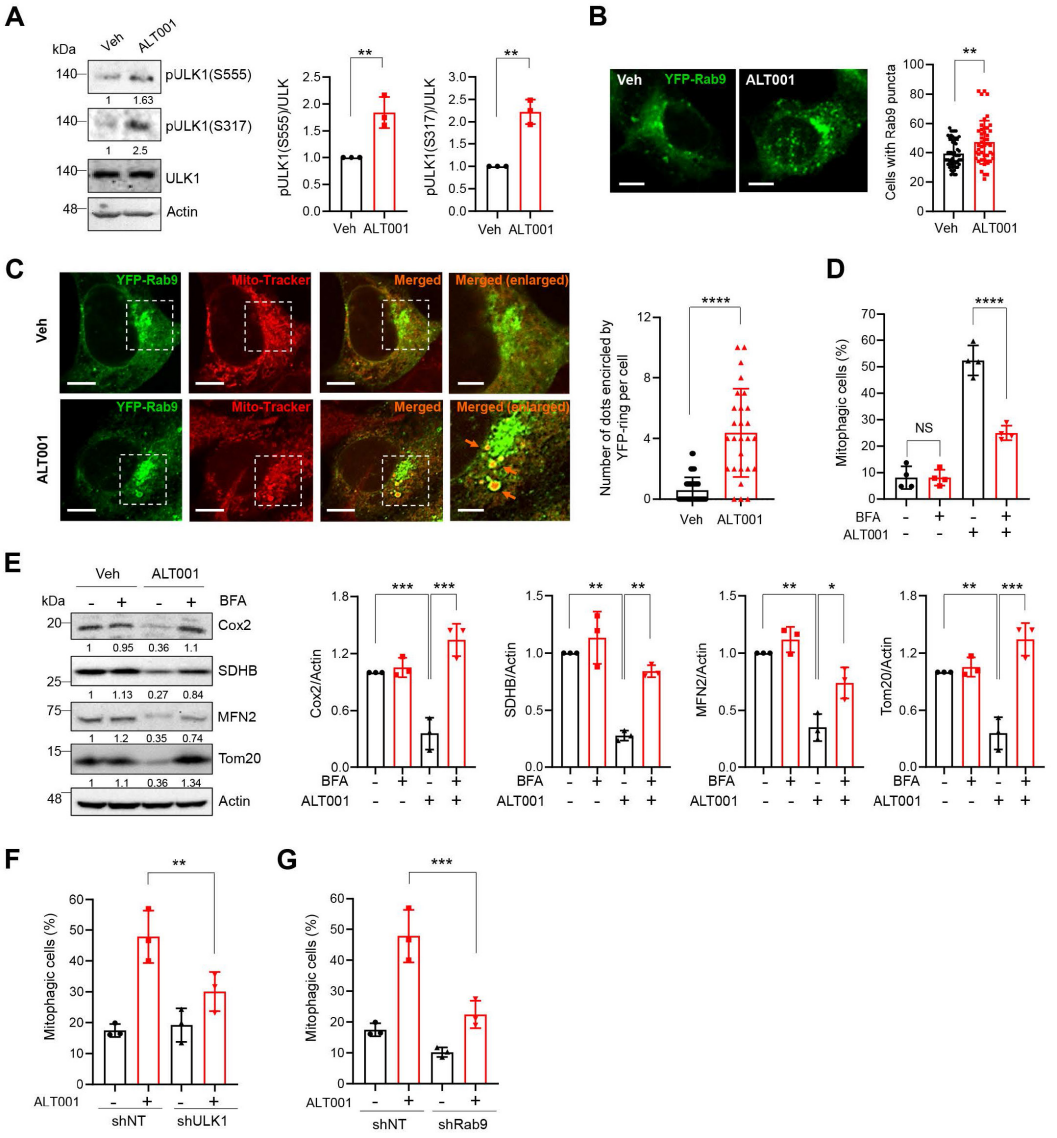
** ALT001 induces mitophagy through the ULK1-Rab9 alternative mitophagy pathway. (A)** BEAS-2B cells were treated with ALT001 (15 μM) for 12 h, and western blot analysis was performed using the indicated antibodies. Quantitative analysis of the protein levels shown on the right as the mean ± SD. **(B)** BEAS-2B cells expressing YFP-Rab9 were treated with ALT001 (15 μM) for 12 h. YFP-Rab9 puncta were analyzed by confocal microscopy, and cells with more than forty YFP-Rab9 puncta were considered positive cells. The percentage of Rab9 puncta-positive cells from three experiments is shown on the right as the mean ± SD (vehicle (Veh), n = 54; ALT001, n = 45). Scale bar: 10 μm. **(C)** BEAS-2B cells expressing YFP-Rab9 were treated with ALT001 (15 μM) for 12 h and stained with MitoTracker Red. Fluorescence images were analyzed by confocal microscopy. Arrows indicate YFP-Rab9-containing mitochondria. Scale bar: 10 μm. The boxed regions are enlarged on the right. Scale bars: 5 μm (right enlarged). The number of YFP-Rab9 puncta-containing mitochondria per cell from three experiments is shown on the right as the mean ± SD (vehicle (Veh), n = 44; ALT001, n = 27). **(D-E)** BEAS-2B cells expressing mt-Keima **(D)** and BEAS-2B cells **(E)** were treated with ALT001 (15 μM) together with brefeldin A (BFA; 1 μg/ml) for 12 h, and mitophagy levels were analyzed by flow cytometry **(D)**. Western blot analysis was performed using the indicated antibodies **(E)**. Quantitative analysis of the protein levels shown on the right as the mean ± SD.** (F-G)** BEAS2B cells expressing shULK1 **(F)** or shRab9 **(G)** were treated with ALT001 (15 μM) for 12 h, and mitophagy levels were analyzed by flow cytometry. The results from three biological replicates **(A, B, F, G)** or four biological replicates **(D)** are shown as the mean ± SD. Significance was determined by Student's *t test* **(A, B, C)** or two-way ANOVA **(D, E, F, G)** with Šidák's multiple-comparison test. **P < 0.01; ***P < 0.001. ****P < 0.0001. Numbers below the corresponding blot represent densitometric analysis normalized to Actin. Blots are representative of three to four biological replicates.

In addition to the canonical PINK1-Parkin pathway, an alternative mitophagy pathway independent of the LC3 and ATG conjugation system was recently identified 17, 18. Activation of unc-51-like kinase 1 (ULK1) and subsequent formation of Rab9-associated autophagosomes have been shown to play central roles in the induction of alternative mitophagy 18. Interestingly, we found that the phosphorylation of ULK1 at Ser555 and Ser317, an indicator of ULK1 activation, was significantly increased upon ALT001 treatment (**Figure 3A**). In addition, ALT001 treatment induced an increase in the percentage of cells containing YFP-Rab9 puncta (**Figure 3B**). To confirm that YFP-Rab9 puncta contain mitochondria, the mitochondrial colocalization of YFP-Rab9 was examined. We found that the number of YFP-Rab9 ring structure-enclosed mitochondria was increased upon ALT001 treatment, indicating that Rab9-associated autophagosomes containing mitochondria were formed upon ALT001 treatment (**Figure 3C**). To verify the role of the ULK1-Rab9-mediated alternative mitophagy pathway, we examined the effect of 16-carbon lactone brefeldin A (BFA), an inhibitor of autophagosome formation in alternative mitophagy 38. BFA treatment significantly inhibited ALT001-induced mitophagy, as indicated by the mt-Keima-based assay (**Figure 3D**), and reduced mitochondrial protein levels, as shown by western blotting (**Figure 3E**). Finally, shRNA-mediated knockdown of ULK1 or Rab9 significantly suppressed ALT001-induced mitophagy (**Figure 3F-G, Figure S4A**). Consistently, the suppression of ALT001-mediated mitophagy upon ULK1 or Rab9 knockdown was also observed through mt-Keima-based assay and western blotting in HEK293 cells (**Figure S4B-F**). These results indicate that ALT001-induced mitophagy is mediated through the ULK1-Rab9-dependent alternative mitophagy pathway.

### ALT001 alleviates mitochondrial dysfunction in an APP Swe/Ind mutant cellular model

**Figure 4:**
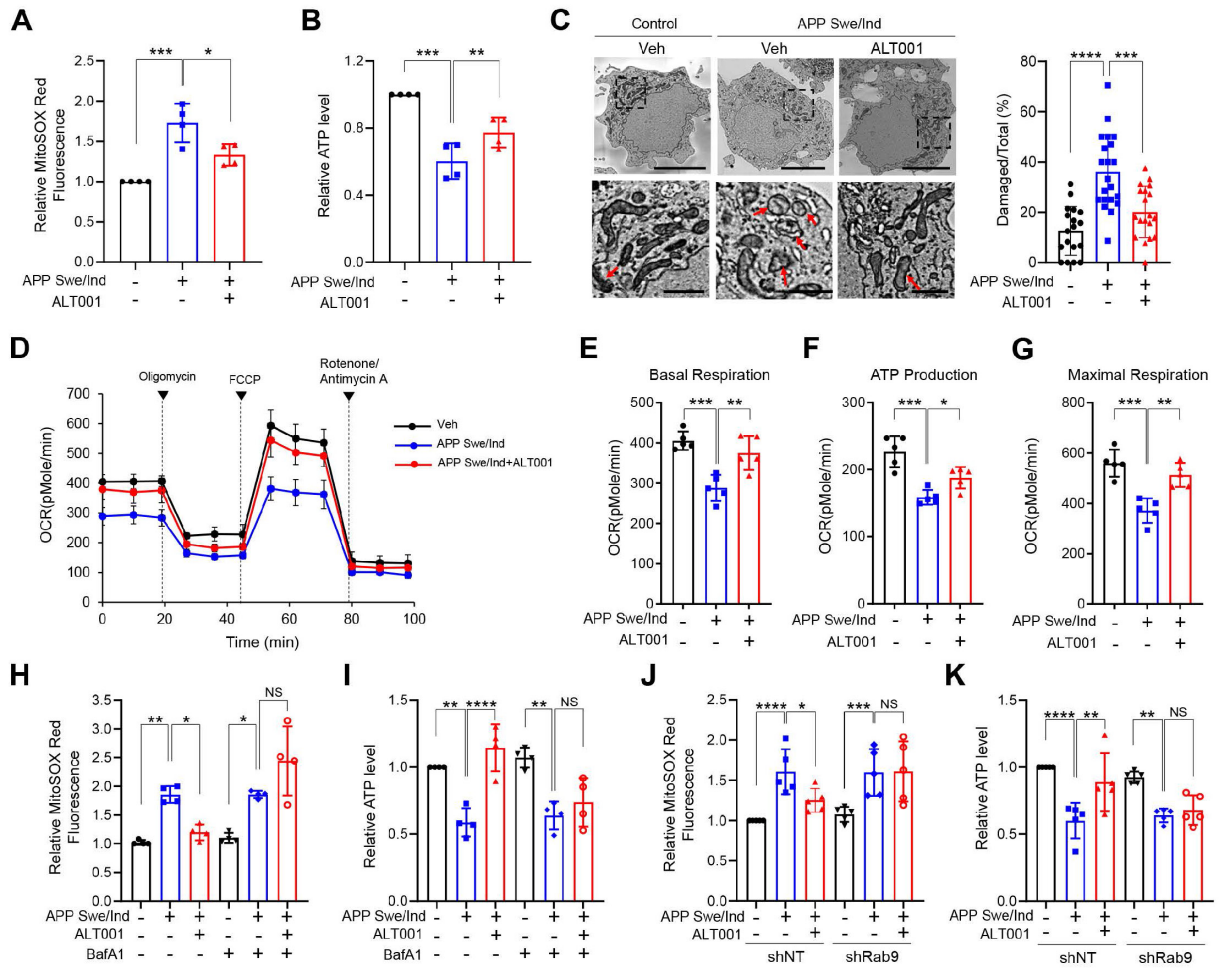
** ALT001 restores mitochondrial function in the APP Swe/Ind mutant cellular model. (A-G)** SH-SY5Y cells expressing the APP Swe/Ind mutant or control plasmid were treated with ALT001 (15 μM) for 12 h. After 48 h of recovery, mitochondrial superoxide levels were measured using MitoSOX Red staining **(A)**, and ATP levels were assessed **(B)**. Cells were analyzed by transmission electron microscopy **(C)**. Arrows indicate damaged mitochondria. Scale bars: 5 μm (upper). The boxed regions are enlarged in the bottom panel. Scale bars: 1 μm (bottom). The proportion of damaged mitochondria per cell is shown on the right as the mean ± SD (control plasmid, vehicle, n = 18; APP Swe/Ind, vehicle, n = 21; APP Swe/Ind, ALT001, n = 19). Representative mitochondrial respiration, analyzed by an XF-24 analyzer with five samples per group, is shown **(D)**. Basal respiration **(E)**, ATP production **(F)**, and maximal respiration **(G)** from mitochondrial respiration analyses are shown as the mean ± SD. **(H-I)** SH-SY5Y cells expressing the APP Swe/Ind mutant or control plasmid (-) were treated with ALT001 (15 μM) alone or cotreated with bafilomycin A1 (BafA1; 100 nM) for 12 h. The mitochondrial superoxide levels were measured using MitoSOX Red staining **(H)**, and ATP levels **(I)** were determined after 48 h of recovery. **(J-K)** SH-SY5Y cells stably expressing shRab9 or control shRNA (shNT) were transfected with APP Swe/Ind mutant or control plasmid and treated with ALT001 (15 μM) for 12 h. After 48 h of recovery, mitochondrial superoxide levels were measured using MitoSOX Red staining **(J)**, and ATP levels were assessed **(K)**. The results from four biological replicates **(A, B, H, I)** or five biological replicates **(D-G, J, K)** are shown as the mean ± SD. Significance was determined by one-way **(A-C, E-G)** or two-way ANOVA (**H-K**) with Šidák's multiple-comparison test. *P < 0.05; **P < 0.01; ***P < 0.001; ****P < 0.0001; NS, not significant.

Recent studies have shown that mitophagy induction exhibits therapeutic potential in AD models 25, 27, 30. To test the effect of ALT001 on AD, we first examined whether ALT001 can ameliorate mitochondrial dysfunction in SH-SY5Y human neuroblastoma cells expressing the APP Swe/Ind mutant, a cellular model of AD, after verification of alternative pathway-dependent mitophagy induction by ALT001 in SH-SY5Y cells (**Figure S5A-F**). We treated SH-SY5Y cells expressing the APP Swe/Ind mutant with ALT001 (15 µM) for 12 h and subsequently examined mitochondrial ROS and ATP production 48 h later to measure the recovery of mitochondrial quality. Ectopic expression of the APP mutant (APP Swe/Ind) in SH-SY5Y cells resulted in an increase in mitochondrial ROS levels and a decrease in ATP production, which are typical changes associated with mitochondrial dysfunction (**Figure 4A-B**). The expression of APP mutants also led to an increase in damaged mitochondria, characterized by disrupted cristae or membranes or loss of matrix density. (**Figure 4C**). Interestingly, ALT001 treatment significantly mitigated the increase in mitochondrial ROS levels, decrease in ATP production and increase in damaged mitochondria (**Figure 4A-C**). We also observed that the decrease in mitochondrial respiration in APP mutant-expressing cells was reversed by ALT001 treatment (**Figure 4D**). All key parameters (basal respiration, ATP production, and maximal respiration) were restored to normal levels upon ALT001 treatment (**Figure 4E-G**).

To verify whether ALT001 exerts its effects through mitophagy, we examined the effect of bafilomycin A1, an inhibitor of autophagosome-lysosome fusion. ALT001-mediated alleviation of mitochondrial dysfunction in APP mutant cells was abolished by bafilomycin A1 (BafA1) (**Figure 4H-I**). Furthermore, Rab9 knockdown suppressed the ALT001-mediated alleviation of mitochondrial dysfunction in APP mutant cells (**Figure 4J-K**). These results suggest that ALT001 relieves mitochondrial dysfunction induced by an APP mutant through Rab9-dependent mitophagy induction.

### ALT001 induces mitophagy *in vivo* and restores mitophagy levels in the AD model

**Figure 5:**
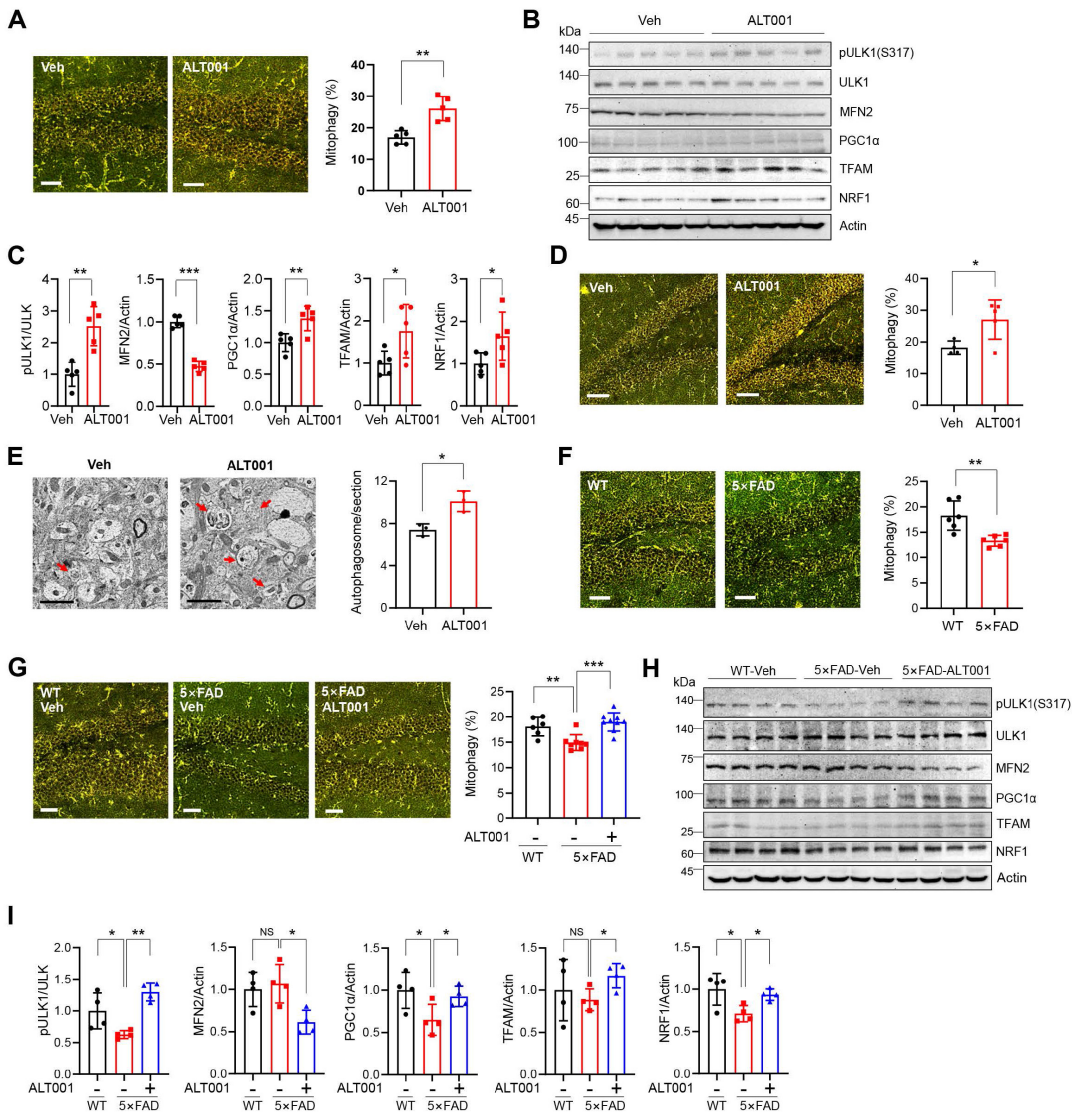
** ALT001 restores the decreased hippocampal mitophagy in the AD mouse model. (A-C)** FVB-mt-Keima male mice were treated with ALT001 (40 mg/kg) or vehicle (Veh) for 4 days via nasal administration (n = 5 per group), and mt-Keima fluorescence was analyzed by confocal microscopy **(A)**. Scale bar: 50 μm. Quantified mitophagy levels from five mice with two images per mouse are shown on the right as the mean ± SD. Hippocampal tissue was isolated, and western blot analysis was performed using the indicated antibodies **(B)**. Quantified protein levels from five mice are shown as the mean ± SD **(C)**. **(D, E)** mt-Keima **(D)** or C57/BL6 male mice **(E)** were treated with ALT001 (1 mg/kg) or vehicle (Veh) for 7 days via nasal administration, and mt-Keima fluorescence was analyzed by confocal microscopy **(D)**. (n = 4 or 5 per group) Scale bar: 50 μm. Quantified mitophagy levels from four mice are shown on the right as the mean ± SD. Autophagosome levels were analyzed by transmission electron microscopy **(E)** (n = 3 per group). Scale bars: 2 μm. Quantified autophagosome levels were determined from several images per mouse and are shown as the mean ± SD. **(F)** mt-Keima fluorescence was analyzed by confocal microscopy in four-month-old wild-type and 5×FAD-mt-Keima male mice (n = 6 per group). **(G-I)** Level of mitophagy in wild-type (WT) and 5×FAD mouse hippocampus upon ALT001 treatment. Four-month-old 5×FAD-mt-Keima male mice were treated with ALT001 (1 mg/kg) or vehicle (Veh) for 7 days via nasal administration, and mt-Keima fluorescence was analyzed by confocal microscopy (**G**). (WT, n = 6; 5×FAD vehicle, n = 8; 5×FAD ALT001, n = 9). Scale bar 50 μm. Hippocampal tissue was isolated, and western blot analysis was performed using the indicated antibodies **(H)**. Quantified protein levels from four mice are shown as the mean ± SD **(I)**. Significance was determined by Student's *t* test **(A, C-F, I)** or one-way ANOVA **(G)** with Šidák's multiple-comparison test. *P < 0.05; **P < 0.01; ***P < 0.001.

To investigate the effect of ALT001 under physiological conditions, we first examined whether ALT001 induces mitophagy *in vivo* using a previously established mt-Keima mitophagy reporter mouse 32. ALT001 efficiently induced mitophagy in primary cortical neurons isolated from mt-Keima mice (**Figure S6A-B**). Then, ALT001 was administered through intranasal administration to deliver it directly to the CNS. Mitophagy assessment in the hippocampus revealed that daily administration of ALT001 (40 mg/kg) for 4 days increased the mitophagy level by approximately 53% (**Figure 5A**). Western blot analysis of the hippocampal region revealed an increase in Ser317 phosphorylation of ULK1, a decrease in MFN2 and an increase in mitochondrial biogenesis factor levels (PGC-1α, TFAM, NRF1) (**Figure 5B-C**), suggesting that ALT001 administration induced the alternative mitophagy pathway and mitochondrial biogenesis simultaneously in the mouse hippocampus. Daily administration of the much lower concentration of ALT001 (1 mg/kg) also increased the mitophagy level after 7 days in the hippocampus by approximately 42% (**Figure 5D**). Western blot analysis also suggested that ALT001 administration at 1 mg/kg increased mitophagy and mitochondrial biogenesis in the hippocampus (**Figure S6C, D**). ALT001 administration also significantly induced mitophagy in the cortex (**Figure S6E**). The number of autophagosomes in the hippocampus was also increased by approximately 36.3% upon ALT001 treatment (1 mg/kg) for 7 days (**Figure 5E**), indicating that ALT001 stimulates mitophagy in the mouse hippocampus.

Recent studies have reported impaired mitophagy in AD mouse models 13, 22-24. Through mitophagy analysis of 4-month-old 5×FAD AD mice crossed with mt-Keima mice, we found that the mitophagy level in the hippocampus of 5×FAD mice was approximately 27% lower than that in wild-type mice (**Figure 5F**). Importantly, the mitophagy level of the hippocampus in 4-month-old 5×FAD mice was restored to that of wild-type mice upon ALT001 administration (1 mg/kg) for 7 days (**Figure 5G**). Western blot analysis revealed that ULK1 phosphorylation levels were reduced in the hippocampus of 5×FAD mice, and we also observed a concurrent decrease in the expression of mitochondrial biosynthesis factors, which is in line with previous reports 23, 39. ALT001 administration increased ULK1 phosphorylation, decreased MFN2, and elevated mitochondrial biogenesis factors (**Figure 5H- I**). These results suggest that ALT001 treatment can restore both mitophagy and mitochondrial biogenesis in the hippocampus of AD mice.

### ALT001 ameliorates cognitive defects and mitochondrial dysfunction in AD models

**Figure 6:**
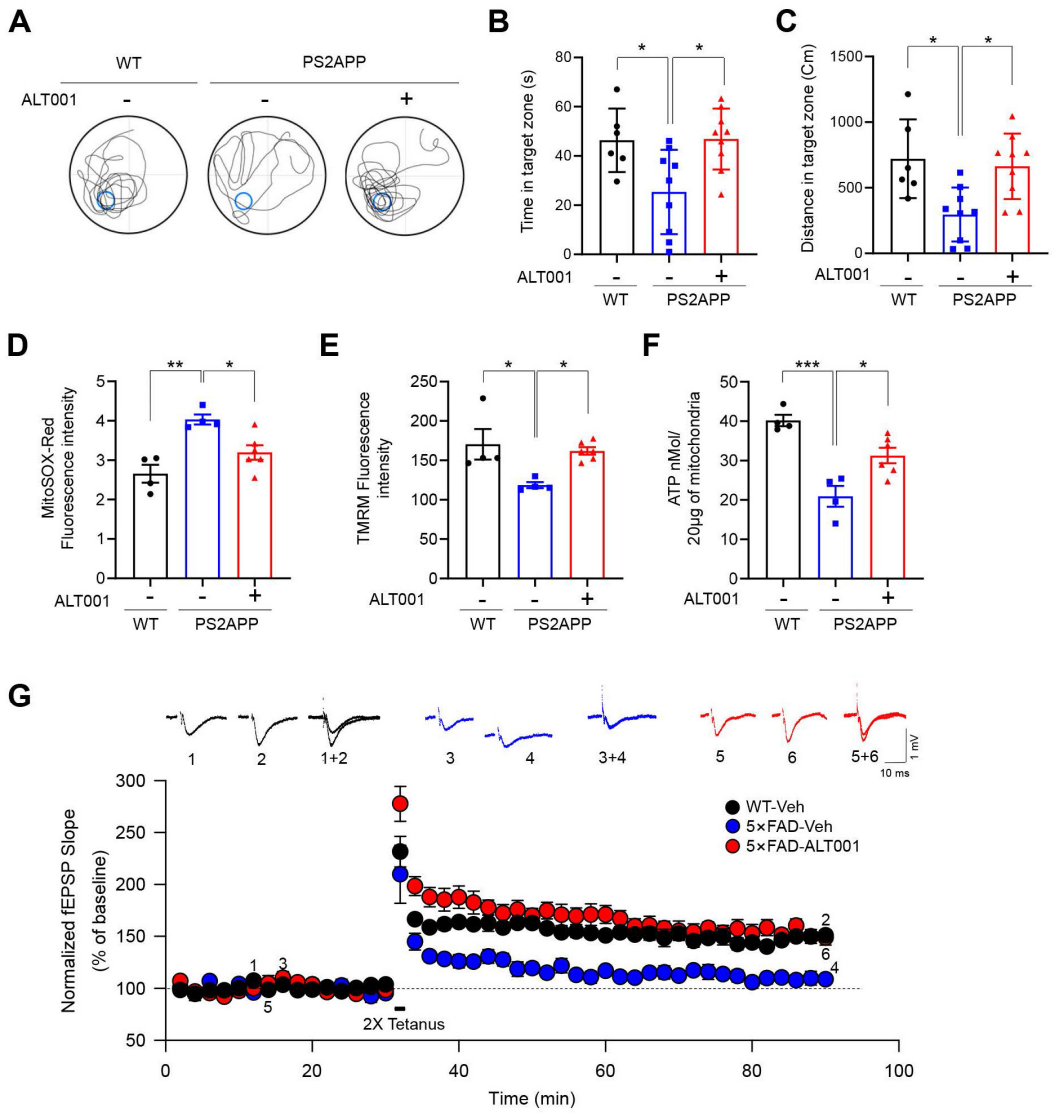
**ALT001 ameliorates cognitive decline and mitochondrial dysfunction in AD mouse models. (A-C)** Ten-month-old PS2APP male mice were treated with ALT001 (1 mg/kg) or vehicle (Veh) via intranasal administration daily for 4 weeks. (WT n = 6, PS2APP + vehicle n = 9, PS2APP + ALT001 n = 9). Representative images of the swimming tracks on day 7 in the Morris water maze test **(A)**. Time spent in the target zone **(B)** and the distance traveled in the target quadrant **(C)** in the probe trial on day 7. The results are shown as the mean ± SD. **(D-F)** PS2APP mice in **(A-C)** were sacrificed after the Morris water maze test, and mitochondria were isolated from the hippocampus. Using the isolated mitochondria, mitochondrial superoxide levels **(D)**, mitochondrial membrane potential **(E)**, and ATP levels **(F)** were analyzed. (WT n = 4, PS2APP + vehicle n = 4, PS2APP + ALT001 n = 6). (**G**) Hippocampal LTP was assessed in four-month-old wild-type treated with vehicle (C57BL/6, black circle, n = 6), 5×FAD mice treated with ALT001 (1 mg/kg) (red circle, n = 6) or 5×FAD mice treated with vehicle (blue circle, n = 5) via intranasal administration daily for 4 weeks. Error bars indicate the SEM. Significance was determined by one-way ANOVA with Šidák's multiple-comparison test. *P < 0.05; **P < 0.01; ***P < 0.001.

To further verify the effect of ALT001 on an AD mouse model, a 10-month-old PS2APP AD mouse model, double transgenic mice expressing mutant PS2 (PS2 N141I) and mutant APP (APP Swe), were also treated with ALT001 daily by intranasal administration (1 mg/kg) for 4 weeks, and cognitive function was assessed using the Morris water maze test. Interestingly, the reductions in the time spent and distance traveled in the target quadrant by PS2APP mice were significantly alleviated upon ALT001 treatment, with ALT001 restoring these parameters to the level observed in wild-type mice (**Figure 6A-C**), suggesting that the impairment of spatial learning and memory ability was relieved upon ALT001 administration in PS2APP mice. Consistently, analysis of mitochondrial function in the hippocampal region in the same group of mice revealed that the increase in mitochondrial ROS levels (**Figure 6D**), decrease in the mitochondrial membrane potential (**Figure 6E**), and decrease in ATP production (**Figure 6F**) were significantly reversed by ALT001 administration.

**Figure 7:**
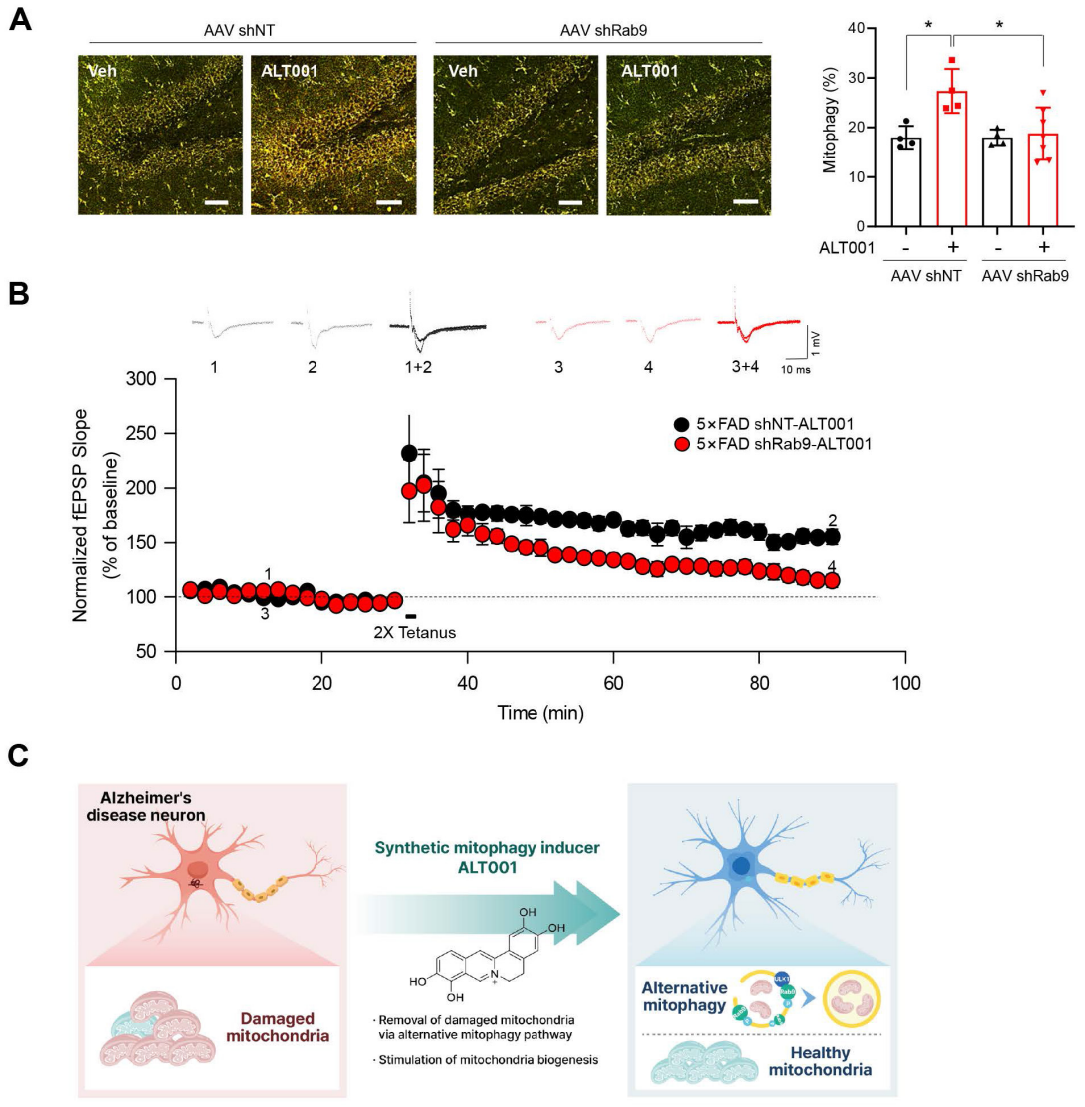
** AAV shRNA-mediated knockdown of Rab9 abolishes ALT001-mediated LTP restoration in 5×FAD mice. (A)** FVB-mtKeima mice were injected with Rab9 shRNA AAV (AAV shRab9) or control shRNA AAV (AAV shNT) into the hippocampus. After 2 weeks, mice were treated with ALT001 (1 mg/kg) or vehicle (Veh) via intranasal administration daily for 7 days, and mt-Keima fluorescence was analyzed by confocal microscopy (AAV shNT vehicle, n = 4; AAV shNT ALT001, n = 4; AAV shRab9 shNT, n = 4, AAV shRab9 ALT001 n = 7). Scale bar: 50 μm. Quantified mitophagy levels from mice are shown on the right as the mean ± SD. (**B**) Four-month-old 5×FAD male mice were injected with Rab9 shRNA AAV (shRab9) or control shRNA AAV (shNT) into the hippocampus. After 2 weeks, the mice were treated with ALT001 (1 mg/kg) via intranasal administration daily for 4 weeks, and LTP analyses were performed (shRab9; red circle, n = 6, shNT; black circle, n = 6). Error bars indicate the SEM. (**C**) Schematic model for ALT001-mediated amelioration of mitochondrial dysfunction in Alzheimer's disease through alternative mitophagy. Significance was determined by one-way ANOVA with Šidák's multiple-comparison test. *P < 0.05.

To further examine the effect of ALT001 on AD, 5×FAD AD model mice were treated with ALT001, and we conducted hippocampal long-term potentiation (LTP) recording, a technique used to study synaptic events occurring during learning and memory 40, 41. It has been shown that hippocampal LTP is impaired in AD mouse models 42, 43. ALT001 was administered through intranasal administration (1 mg/kg) daily for 4 weeks. Consistent with previous reports, LTP analysis in hippocampal brain slices showed that synaptic function was significantly impaired in 5×FAD mice (5×FAD -Veh, 108±8%, n = 5) compared to wild-type mice (WT, 150±5%, n = 6). Furthermore, the one-month intranasal administration of ALT001 significantly rescued LTP in 5×FAD mice (5×FAD-ALT001, 148±7%, n = 6) (**Figure 6G**). Furthermore, the restoration of decreased LTP was also observed in Tg2576, another well-known AD mouse model (**Figure S7**). These results suggest that ALT001 treatment improved synaptic function in AD model mice. To verify whether the therapeutic effect of ALT001 on LTP recovery in 5×FAD mouse is dependent on alternative mitophagy, we performed knockdown of Rab9 in the mouse hippocampus using AAV-mediated delivery of Rab9 shRNA. Stereotactic injection of Rab9 shRNA (shRab9) AAV virus into the hippocampus significantly reduced the expression of Rab9 and ALT001-mediated mitophagy induction (**Figure S8, Figure 7A**). Consistent with a previous report 18, mitophagy levels in control mice were not changed by Rab9 knockdown (**Figure 7A**). Importantly, the injection of shRab9 AAV abolished the restoration of LTP by ALT001 treatment in 5×FAD mice (5×FAD-shRab9-ALT001, 114±5%, n = 6), whereas the control AAV-injected, ALT001 one-month intranasally administered 5×FAD mice showed a significant rescue of LTP (5×FAD-shNT-ALT001, 155±6%, n = 6) (**Figure 7B**). These results suggest that ALT001 alleviates cognitive impairment in AD mouse model through the induction of alternative mitophagy pathway (**Figure 7C**).

## Discussion

The development of an effective mitophagy inducer with low toxicity and a defined molecular mechanism is critical for the clinical application of mitophagy-based therapies 14. In this study, we identified an isoquinolium scaffold for mitophagy induction through chemical library screening using a mitophagy reporter system and generated ALT001 through chemical optimization of the isoquinolium scaffold. By comparing mitophagy activity using mt-Keima-based quantitative analysis, we found that ALT001 resulted in approximately 25-fold higher mitophagy activity than the parent chemical palmatine. ALT001 selectively induces mitochondrial degradation and subsequent mitochondrial biogenesis. ALT001 efficiently induces mitophagy but does not change the intracellular levels of other organelles. These results suggest that ALT001 is an efficient mitophagy inducer. Our study also suggests that mitophagy activity itself is a better readout in library screening to identify mitophagy inducers than other indirect readouts, such as LC3 puncta formation or Parkin translocation.

One of the primary considerations for using mitophagy as a treatment for diseases is the development of mitophagy inducer compounds that have low toxicity 14, 31. Mitophagy inducers widely used in mitophagy research, such as CCCP, are restricted in their therapeutic use due to toxicity 34. We also identified actinonin as a mitophagy inducer 32, but the high toxicity of actinonin made it impossible to use in animal models (data not shown). ALT001 does not induce changes in the mitochondrial membrane potential in cellular treatment. In addition, we verified that ALT001 induces mitophagy in mouse primary cortical neurons and hippocampal and cortical tissues* in vivo*. These results suggest that ALT001 is suitable for mitophagy-based therapeutic strategies.

Identification of the molecular mechanism is also important for the clinical application of mitophagy inducers 14, 44. In this study, we identified that the ULK-Rab9-dependent alternative mitophagy pathway is responsible for ALT001-induced mitophagy independent of the canonical mitophagy pathway. Unlike the PINK1-Parkin canonical mitophagy pathway, the alternative mitophagy pathway is not dependent on LC3 and ATG7 45. While the membrane of autophagosomes formed via the canonical pathway is derived mainly from the ER, Golgi apparatus-derived membranes play a central role in the alternative mitophagy pathway 46. Thus, alternative mitophagy is inhibited by brefeldin A, an inhibitor of vesicle transport from the Golgi apparatus 18, 38, while canonical mitophagy is not. Consistent with these studies, we found that ALT001-induced mitophagy is not dependent on LC3, ATG7, or PINK1. ATL001 efficiently induced mitophagy in ATG7 knockout cells and in PINK1 knockdown cells, while CCCP-induced mitophagy was suppressed. In contrast, ALT001 treatment induced the activation of ULK1 and the mitochondrial localization of Rab9. Moreover, inhibition of ULK1 or Rab9 abolished ALT001-induced mitophagy, indicating that the ULK1-Rab9-dependent alternative mitophagy pathway is responsible for ALT001-induced mitophagy. A recent study showed that alternative mitophagy plays a critical role in the protection of cardiac function under various stress conditions. Rab9-dependent mitophagy is responsible for mitochondrial clearance in cardiomyocytes upon IGF-II treatment 47. Saito et al showed that mitophagy occurs through the ULK1-Rab9-dependent alternative pathway in cardiomyocytes under energy stress conditions 18. More recently, Tong et al reported that ULK1-Rab9-dependent alternative mitophagy mediates the clearance of damaged mitochondria under high-fat diet feeding and plays a critical role in cardiac protection 48, 49. These results indicate that the ULK1-Rab9 alternative pathway plays an essential role in maintaining heart function. Our study showed that activation of the alternative pathway by ALT001 rescues mitochondrial dysfunction resulting from mutant APP expression. Furthermore, ALT001-induced mitophagy restored mitophagy levels and mitochondrial biogenesis in 5×FAD mice. ALT001 also restored mitochondrial function in the hippocampus in PS2APP model mice. These results suggest that the ULK1-Rab9-dependent alternative mitophagy pathway plays a critical role in mitochondrial homeostasis in brain tissue in addition to the heart. Given that the heart and brain are highly energy-demanding organs, the results from our group and other groups suggest that alternative pathways could be more important, especially in mitochondria-dependent organs.

Notably, the level of ULK1 was not changed upon ALT001 treatment, while an increase in ULK1 has been shown to stimulate alternative mitophagy under different stress conditions 18, 45. These differences suggest that ALT001 could activate the alternative mitophagy pathway through a different mechanism than previously reported. The exact molecular mechanism underlying ALT001-mediated activation of the alternative mitophagy pathway should be clarified in further studies. Regarding how ALT001 activates the ULK1-Rab9 alternative mitophagy pathway, we are currently investigating the direct binding target of ALT001. Identification of ALT001-binding proteins would provide valuable information about the exact molecular mechanism of ALT001 and facilitate the development of more specific mitophagy inducers for the activation of alternative mitophagy in AD.

Recent studies have reported that stimulation of mitophagy is beneficial for ameliorating cognitive impairment as well as mitochondrial dysfunction 25, 27, 30. Naturally occurring compounds such as NR and urolithin A have also been shown to enhance mitophagy and to improve deficits in AD models 28, 29. These studies suggest that mitophagy induction through multiple pathways could be beneficial for AD treatments. Consistent with these studies, we observed that ALT001 administration ameliorated cognitive defects in the PS2APP mouse model. Furthermore, we observed that ALT001 rescued defects in long-term potentiation in the 5×FAD and Tg2576 AD mouse models, suggesting that ALT001 has potential beneficial effects on AD models. We further verified the mitophagy-dependent therapeutic effect of ALT001 through AAV-mediated knockdown of Rab9 in the hippocampus. Recent studies have shown that stimulation of mitophagy also inhibits major pathological processes, such as the accumulation of Aβ protein, the hyperphosphorylation of Tau, and neuroinflammation 25, 30. Whether ALT001 administration also inhibits these AD pathologies remains to be further explored.

Taken together, our results identify ALT001 as a potential mitophagy inducer with low toxicity, which is required for mitophagy-based treatment of human diseases. In addition, the results of the present study propose for the first time that the ULK1-Rab9-dependent alternative mitophagy pathway is a promising target for AD treatment. Although the precise molecular mechanisms should be further explored in additional studies, the results of our study raise the possibility of the clinical application of mitophagy-based therapeutics for AD.

## Materials and Methods

### Cell lines, plasmids and treatments

BEAS-2B, SH-SY5Y, HEK293 and HeLa cells were maintained in DMEM containing 10% fetal bovine serum (FBS; JR Scientific Inc., Woodland, CA, USA). HeLa and ATG7 knockout (KO) HeLa cells were kindly provided by Dr. Tomotake Kanki (Niigata University Graduate School of Medical and Dental Sciences). ATG7 KO HeLa cells stably expressing Flag-Parkin were generated by transfection of pcDNA3-Flag-Parkin (kindly provided by Dr. Jongkyeong Chung of Seoul National University) DNA and neomycin selection. Cell lines stably expressing mt-Keima or Keima were generated by infection with a lentivirus produced by using a pLVX-mtKeima or pLESIP-Keima lentiviral construct 32.

A YFP-Rab9 expression construct (pDC316-YFP-Rab9) was kindly provided by Dr. Junichi Sadoshima of Rutgers New Jersey Medical School. EGFP-LC3 (#11546), pLV-ER GFP (#80069), pLV-Golgi eGFP (#79809), pmTurquoise2-Peroxi (#36203), and pCAX APP Swe/Ind (#30145) were obtained from Addgene (Watertown, MA, USA). A mitochondrial YFP-expressing plasmid (pLESIP-mitoYFP) was generated by subcloning mitoYFP from pcDNA3-mitoYFP (provided by Dr. Gyesoon Yun, Ajou University) into the pLESIP vector. Carbonyl cyanide 3-chlorophenylhydrazone (CCCP) and bafilomycin A1 were purchased from Sigma‒Aldrich (St. Louis, MO, USA). Brefeldin A (BFA) was obtained from Selleckchem (Houston, TX, USA). MitoTracker Red CMXRos was purchased from Invitrogen (Carlsbad, CA, USA).

### Chemical library screening using the mt-Keima assay system

Sixty-one individual compounds were dissolved in DMSO and administered to BEAS-2B-mt-Keima cells at the indicated concentrations (**Table S1**). Dimethyl sulfoxide (DMSO) was used as a negative control, and CCCP (10 μM) was used as a positive control. Mitophagy activity was determined using an LSR Fortessa flow cytometer (BD Biosciences, Franklin Lakes, NJ, USA) equipped with 405 nm and 561 nm lasers at the Neuroscience Translational Research Solution Center (Busan, South Korea) as described previously 33. Briefly, we first drew a gate around untreated HeLa-mt-Keima cells. The percentage of cells undergoing mitophagy (mitophagic cells (%)) was determined by gating cells exhibiting a high ratio of emission at 561 nm/405 nm excitation. To distinguish between high and low ratios of emission at 561 nm/405 nm excitation, we used untreated HeLa-mt-Keima cells exhibiting low mitophagy activity as a standard for a low ratio 33.

### Chemical synthesis of isoquinoline derivatives

Six isoquinolinium derivatives (**1**-**6**) were synthesized from palmatine and berberine (Sigma‒Aldrich), as shown in **Figure S1**. First, the selective removal of the methyl group on the C-9 position of palmatine and berberine at 200 ± 10 °C under high vacuum conditions resulted in the synthesis of compounds **1** and **2** with one hydroxy group in yields of 74% and 82%, respectively. The reaction of **2** with BCl_3_ removed a methylene group to generate compound **5** in a yield of 91%. The treatment of berberine with BCl_3_ gave corresponding isoquinolinium analog **4** in a yield of 94%. Finally, all compounds, including berberine, palmatine, and their intermediates, were converted into a unique isoquinolinium structure (**3**) with four hydroxyl groups in quantitative yield by treatment with BBr_3_. Subsequently, the esterification of **3** with acyl chloride, a ubiquitous strategy to increase the efficacy or efficiency of a drug candidate for drug discovery, produced a corresponding ester derivative (**6**), as a prodrug form of **3**, in a yield of 87%.

### Measurement of mitophagy and autophagy levels using confocal microscopy

To measure mitophagy levels in cells and tissue samples, mt-Keima fluorescence was examined using a Zeiss LSM 700 confocal microscope equipped with a C-Apochromat 40x/1.20 W Korr M27 lens at the Neuroscience Translational Research Solution Center (Busan, South Korea). mt-Keima fluorescence was imaged using two sequential excitation lasers (458 nm and 561 nm) and a 595-700 nm emission bandwidth. We depicted the mt-Keima fluorescence signal from the 458 nm excitation wavelength in green and the signal from excitation by the 561 nm laser in red. Quantitation of mitophagy levels based on mt-Keima confocal images was performed using Zeiss Zen software as described previously 32, 50. The mitophagy level (mitophagy (%)) was defined as the number of pixels with a high red/green ratio divided by the total number of pixels.

To quantify the mitophagy level in cells, the experiment was independently repeated three times, and at least five images per sample were analyzed in each experiment. To quantify the mitophagy level in mouse tissue, mt-Keima fluorescence was analyzed in at least four mice, with two images per mouse being examined. To measure autophagy levels in cells, Keima fluorescence was analyzed, and the level of autophagy (autophagy (%)) was determined in the same manner as the level of mitophagy described above. In all confocal microscopy analyses, all imaging parameters remained constant, and only the gain level was adjusted to avoid saturation of any pixel. All mitophagy and autophagy measurement results are presented as the mean ± SD.

### Analysis of cell death

BEAS-2B cells were treated with CCCP (10 μM) or ALT001 (15 μM) for 24 h and incubated for 3 days. Cell death was assessed using a FITC Annexin V Apoptosis Detection Kit 1 (BD Biosciences, Franklin Lakes, NJ, USA) according to the manufacturer's protocol. The cells were analyzed using an Attune NxT cytometer (Thermo Fisher Scientific, Waltham, MA, USA) and Attune NxT Software (Thermo Fisher Scientific) was used to analyze the cell apoptosis rate. The experiment was independently repeated three times, and the results are presented as the mean ± SD.

### Western blot analysis and antibodies

Cells were lysed in RIPA buffer and subjected to western blot analysis as described previously 51. Anti-Cox2 (ab198286), anti-MFN2 (ab56889), anti-SDHB (ab14714), anti-P4HB (ab137110), anti-PMP70 (ab3421), anti-PGC-1α (ab191838), anti-TFAM (ab272885), and anti-NRF1 (ab175932) antibodies were purchased from Abcam (Cambridge, UK). An anti-LC3B (#L10382) antibody was purchased from Invitrogen (Waltham, MA, USA). Anti-ULK1 pS555 (#5869), anti-ULK1 pS317 (#37762), anti-ULK1 (#8054), and anti-GM130 (#12480) antibodies were purchased from Cell Signaling Technology (Danvers MA, USA). An anti-OPA1 (#612606) antibody was purchased from BD Biosciences (San Jose, CA, USA). An anti-PINK1 (#BC100-494) antibody was purchased from Novus Biologicals (Littleton, CO, USA). Anti-Tom20 (SC-11415) and anti-Actin (SC-47778) antibodies were purchased from Santa Cruz (Dallas, TX, USA). All western blot analyses were repeated three times. Band intensities were quantified using densitometry and ImageJ software (NIH).

### Electron microscopy analysis

Ultrathin sections of cells and mouse tissues for electron microscopic analysis were prepared at the Neuroscience Translational Research Solution Center at Dong-A University. Ultrathin sections were observed and photographed using a Talos transmission electron microscope (Thermo-Fisher Scientific, Waltham, MA, USA) or an Apreo 2S LoVac scanning electron microscope (Thermo-Fisher Scientific) at the Neuroscience Translational Research Solution Center, Dong-A University.

For analysis of autophagosome formation upon ALT001 treatment, BEAS-2B cells treated with ALT001 were analyzed by transmission electron microscopy. Twenty cells per group were examined to determine the number of autophagosomes containing mitochondria per cell. For analysis of the damaged mitochondria upon APP mutant expression, the proportion of damaged mitochondria, characterized by disrupted cristae or membrane or loss of matrix density, was determined in SH-SY5Y cells expressing the APP Swe/Ind mutant. At least eighteen cells per group were examined to determine the proportion of damaged mitochondria in each group. For analysis of autophagosome formation upon ALT001 treatment in the mouse hippocampus, the hippocampal tissues of C57/BL6 male mice treated with ALT001 were analyzed by transmission electron microscopy. The number of autophagosomes containing mitochondria was determined by analyzing several images from each mouse, and the results are presented as the mean ± SD.

### Measurement of mitochondrial membrane potential and mitochondrial ROS

Mitochondrial membrane potential was measured using tetramethylrhodamine methyl ester (TMRM) (Invitrogen), and mitochondrial ROS levels were measured using MitoSOX Red (5 μM) (Invitrogen, Carlsbad, CA) as described previously 51. The TMRM fluorescence intensity and MitoSOX Red fluorescence intensity were analyzed by flow cytometry using an LSR Fortessa cytometer.

### Measurement of mitochondrial mass

Mitochondrial mass was measured by using 10-*N*-nonyl-acridine orange (NAO) (1 μM) (Cayman, Ann Arbor, MI) staining. The NAO fluorescence intensity was measured by confocal microscopy using a Zeiss LSM 700 confocal microscope.

### Confocal microscopy of fluorescent organelle-specific markers

To analyze the level of organelles upon ATL001 treatment, cells expressing fluorescent markers for the endoplasmic reticulum (ER: pLV-ER GFP), Golgi (pLV-Golgi eGFP), peroxisome (pmTurquoise2-Peroxi) or mitochondria (mito-YFP) were examined using a Zeiss LSM 700 confocal microscope at the Neuroscience Translational Research Solution Center. To determine the fluorescence intensities of organelle-specific markers, at least five images per sample were analyzed. The experiment was independently repeated three times, and the results are presented as the mean ± SD.

### Analysis of LC3 and Rab9 puncta

For LC3 puncta analysis, EGFP-LC3 puncta were analyzed by confocal microscopy. At least thirty cells per group in three repeated experiments were examined, and EGFP-LC3 puncta were counted. The results (EGFP-LC3 puncta/cell) are presented as the mean ± SD.

For Rab9 puncta analysis, YFP-Rab9 puncta were analyzed by confocal microscopy, and cells containing more than forty YFP-Rab9 puncta were considered positive cells. At least forty-five cells per group in three repeated experiments were examined, and YFP-Rab9 puncta-positive cells were counted. The results (cells with Rab9 puncta (%)) are presented as the mean ± SD.

For the analysis of the mitochondrial localization of YFP-Rab9, cells expressing YFP-Rab9 were treated with ALT001 and stained with 100 nM MitoTracker Red for 30 min. The number of YFP-Rab9 ring structure-enclosed mitochondria per cell was counted by confocal microscopy. To quantify the mitochondrial localization of YFP-Rab9, at least twenty-five cells per group in three repeated experiments were examined, and the results are presented as the mean ± SD.

### shRNA-mediated knockdown

To knockdown PINK1 and Rab9, lentiviral constructs containing PINK1, Rab9 shRNA (shPINK1; TRCN0000199193, shRab9a; TRCN0000048105) and control nontargeting shRNA (shNT; SHC016) were obtained from Sigma Aldrich. For ULK1 knockdown, lentiviral constructs containing ULK1 shRNA (#27633) were obtained from Addgene. Knockdown shRNA constructs were transfected into 293FT packaging cells, and the resulting cell-free viral supernatant was used to infect cells. After puromycin selection, resistant cells were pooled and used for the remaining experiments.

### Measurement of ATP levels

The levels of intracellular ATP were measured using the ENLITEN® ATP Assay System (Promega, Madison, WI, USA). Cell lysates were prepared by subjecting the cells to three cycles of freezing and thawing using ATP extraction buffer (100 mM Tris, pH 7.6, 4 mM EDTA). ATP content in the cell lysates was analyzed according to the manufacturer's recommendations. Bioluminescence values were measured using a LuBi Microplate Luminometer (MicroDigital Co., Seongnam, Korea) and normalized to the protein concentration in the corresponding lysates. The experiment was repeated four to five times, and the results are presented as the mean ± SD. For the analysis of ATP levels in the mouse hippocampus, isolated mitochondria (20 μg) were resuspended in ATP extraction buffer and analyzed using the same ATP assay system. ATP content was analyzed in four to six mice per group, and the results are presented as the mean ± SD.

### Mitochondrial respiration analysis

Cellular respiration rates were measured in a 24-well plate using an XF24 flux analyzer (Seahorse Bioscience Inc. North Billerica, MA, USA) as described previously. 51 The oxygen consumption rate was measured under basal conditions followed by the sequential addition of oligomycin (0.5 μM), carbonyl cyanide p-trifluoromethoxaminehydrazone (FCCP, 1 μM), and rotenone (1 μM)/antimycin A (1 μM) to assess basal respiration, proton leakage, maximal respiration, nonmitochondrial respiration and ATP production. The oxygen consumption parameters were normalized to the number of cells. The experiment was repeated five times, and the results are presented as the mean ± SD.

### Animal experiments

FVB-mt-Keima mice were generated previously 32. 5×FAD mice (APP KM670/671NL [Swedish], APP I716V [Florida], APP V717I [London], PSEN1 M146L, PSEN1 L286V) were obtained from Jackson Laboratory (Bar Harbor, ME, USA). PS2APP model mice (C57BL/6-Tg(NSE-hPS2*N141I);Tg(NSE-hAPP Swe)) were obtained from the National Institute of Food and Drug Safety Evaluation (NIFDS, Cheongju, Korea), and all procedures were performed according to a protocol approved by the Dong-A Institutional Animal Care and Use Committee (DIACUC-22-19). For the electrophysiology experiments, 5×FAD and Tg2576 (APP KM670/671NL, Taconic) mice were used, and experiments were performed according to a protocol approved by the Institutional Animal Care and Use Committee of Chonnam National University Medical School. To generate 5×FAD-mt-Keima mice, 5×FAD mice were crossed with FVB-mt-Keima mice.

For the analysis of mitophagy induction upon ALT001 treatment, three to 4-month-old FVB-mt-Keima mice or 5×FAD-mt-Keima mice were intranasally administered 40 mg/kg ALT001 dissolved in solution (50% DMSO, 10% Tween 80) daily for four days or 1 mg/kg ALT001 daily for seven days. For the analysis of the therapeutic effect of ALT001 on the AD mouse model, 5×FAD, PS2APP and Tg2576 mice were intranasally administered 1 mg/kg ALT001 dissolved in solution (5% DMSO, 10% Tween 80) daily for four weeks from 4, 10 and 9 months of age. All animal experiments were performed in a blinded fashion.

### Primary cortical neuron isolation

Primary mouse cortical neurons were prepared as previously described 52. Briefly, cerebral cortices were isolated from FVB-mt-Keima mouse embryos on the 14th embryonic day. The neurons were resuspended in neurobasal medium (Gibco #21103049) supplemented with 2% B27 (Gibco #17504044) and 0.25% GlutaMax (Gibco #35050061) and seeded in confocal dishes coated with 0.01% poly-D-lysine (Sigma #P0899). Half of the medium was replaced every 3 days. The neurons were used at 10 days *in vitro* for subsequent experiments.

### Morris water maze

The Morris water maze test was performed to analyze long-term learning and spatial memory as described previously 53, 54. A circular target platform (10 cm diameter) was immersed in a pool (diameter of 120 cm, depth of 50 cm), and a high-contrast cue was attached to the inside of the pool near the platform above the water surface. The test was conducted every 24 hours for 7 consecutive days. Before starting the main experiment, all mice were allowed to swim in the presence of the cue, and a 90-second visible platform trial was performed on day 1 to allow adaptation to water. On days 2-6, the mice were placed in the water with their heads facing the wall of the pool. In the hidden platform trials, the water was made opaque, and the mice were placed in the pool four times from different quadrants for 5 consecutive days. On day 7, the platform was removed from the pool, and the probe trial was performed for 90 seconds. The swimming trajectories were video-recorded. The distance traveled and time spent in the quadrant containing the platform were measured using Smart software (Panlab, Barcelona, Spain). The experimenter was blinded to the treatment of the animals and the data analysis.

### Hippocampal mitochondria isolation

Mouse hippocampal mitochondria were isolated according to a previously established method with modifications 6, 55. Briefly, mouse brains were quickly removed and washed with ice-cold PBS, and the hippocampus was isolated from the left hemisphere. The final mitochondrial pellet was suspended in isolation buffer 3 containing 215 mM mannitol, 75 mM sucrose, 0.1% BSA, and 20 mM HEPES (pH 7.2) to yield a final protein concentration of approximately 1 mg/ml and immediately stored on ice. Twenty micrograms of mitochondria were used for TMRM staining, MitoSox Red staining and an ATP assay.

### Hippocampal slice preparation and electrophysiology

Mouse hippocampal slice preparation and electrophysiology experiments were conducted as previously described 40, 41. The experiment was conducted between 9:30 and 10:00 a.m. The animals were sacrificed by cervical dislocation, and the brain was quickly removed and transferred to ice-cold artificial cerebrospinal fluid. The brain was cut midsagittally, and one hemisphere was returned to ice-cold artificial cerebrospinal fluid (aCSF) until analysis. Hippocampal slices were prepared by transverse sectioning (400 µm thick) using a McIlwain tissue chipper (Mickle Laboratory Engineering Co. Ltd., UK).

After recovery for approximately 60 min after the slice preparation procedure, electrophysiology experiments were conducted. Extracellular field potentials were recorded in the CA1 region using microcapillary electrodes. After establishing a stable baseline for 30 min, LTP was evoked by two trains of high-frequency tetanic stimuli (each 100 Hz, 1 s: repeated after a 30-s interval), field excitatory postsynaptic potentials (fEPSPs) were recorded for at least 60 min, and the slope of the evoked fEPSP response was measured and is expressed relative to the preconditioning baseline data. Data were collected by an NI USB-6251 data acquisition module (National Instruments, Texas, USA), amplified by an Axopatch 200B amplifier (Axon Instruments, CA, USA), captured and analyzed by WinLTP Software. The experimenter was blinded to the treatment of the animals and the data analysis.

### Stereotactic injection of Rab9 shRNA adeno-associated virus (AAV)

The AAV2-CFP-U6-m Rab9 shRNA and AAV2-CFP-U6-scrmb-shRNA viruses were produced by Vector BioLabs. (Malvern, PA, USA). To inject AAV, mice were anesthetized and placed in a stereotactic frame (Stoelting Digital Stereotaxic Instrument, Wood Dale, IL, USA). 5×FAD and FVB-mt-Keima mice at 4 months of age were injected into the hippocampus (AP:-2 mm, M/L: 1.5 mm, DV:1.75 mm) with 2 x 10^9^ viral particles per mouse at a rate of =0.2 μl per min. After 2 weeks, mice were intranasally administered 1 mg/kg ALT001 dissolved in solution (5% DMSO, 10% Tween 80).

### Statistical analysis

All data are presented as the mean ± SD. Differences between two experimental groups were analyzed using Student's *t test*. To compare three or more groups, we used one-way or two-way ANOVA with Sidák's correction. A *P value* < 0.05 was considered statistically significant.

## Supplementary Material

Supplementary figures and table.
