# Supplementary material for: Selective induction of Rab9-dependent alternative mitophagy using a synthetic derivative of isoquinoline alleviates mitochondrial dysfunction and cognitive deficits in Alzheimer's disease models
Source: Theranostics. 2024 Jan 1;14(1):56–74. doi: 10.7150/thno.88718 (PMC10750208; doi:10.7150/thno.88718)
Supplement: Supplementary file 1 — Supplementary figures and table. [file thnov14p0056s1.pdf]

Supplementary materials

Selective induction of Rab9-dependent alternative mitophagy using a synthetic derivative of isoquinoline alleviates mitochondrial dysfunction and cognitive deficits in Alzheimer's disease models

Jee-Hyun Um[1,2*], Dong Jin Shin[1,2,3*], Se Myeong Choi[4*], Alen Benhur Pravin Nathan[5*], Young Yeon Kim[1,2,3], Da Ye Lee[1,2,3], Dae Jin Jeong[1,2,3], Dong Hyun Kim[6], Kyung Hwa Kim[7], Young Hye Kim[8], Jihoon Nah[9], Jeong-hee Jeong[10], Eunhee Yoo[10], Hwa Kyoung Shin[11], Hwan Tae Park[1,3,12], Jihoon Jo[5], Jong Hyun Cho[3,4] and Jeanho Yun[1,2,3]

Table S1.

number	Chemical name	treatment concentration	% of mitophagy	viability
	control		8.5	100
	CCCP	20 μM	88.5	70
1	Daidzein	250 μM	7.5	100
2	Gallic acid	50 μM	35.7	100
3	β-sitosterol	5 μM	8.3	100
4	Timosaponin A-Ⅲ	5 μM	15.5	100
5	Genistein	250 μM	6.5	100
6	Tanshinone 2a	50 μM	22.0	100
7	Palmatine	500 μM	91.5	70
8	Puerarin	250 μM	6.1	100
9	Daidzin	250 μM	6.7	100
10	Pinoresinol	250 μM	6.9	100
11	Mangiferin	50 μM	11.6	100
12	Saikosaponin D	5 μM	9.7	100
13	Cryptotanshinone	12.5 μM	18.3	100
14	Curcumin	5 μM	10.5	100
15	Luteolin-7-O-glucuronide	50 μM	7.9	100
16	Salvianolic acid B	10 μM	8.3	100
17	(+)-ar-turmerone	500 μM	12.0	70
18	Swertiamarin	500 μM	8.4	100
19	Dammarenediol Ⅱ	250 μM	24.6	100
20	Tanshinone Ⅰ	25 μM	10.3	100
21	epi-berberine	250 μM	90.9	70
22	Berberine	250 μM	46.6	80
23	Swertisin	100 μM	7.0	100
24	Spinosin	50 μM	9.8	100
25	Limonin	25 μM	9.9	100
26	Ferulic acid	250 μM	11.9	100
27	4-hydroxy-benzoic acid	500 μM	11.2	100
28	triterpenoid glycoside	50 μM	26.0	80
29	Jujuboside B	100 μM	29.1	100
30	Loganin	500 μM	10.3	100
31	Magnolin	50 μM	16.8	100
32	Eugenol	500 μM	30.4	100
33	Obacunone	500 μM	16.1	100
34	Scoparone	500 μM	41.8	80
35	Rutaecarpine	250 μM	17.2	100
36	Umbelliferone	500 μM	8.2	100
37	Soyasaponins	10 μM	12.7	100
38	P-coumaric acid	500 μM	12.0	100
39	Rhoifolin	50 μM	13.9	100
40	Schisandrol A	100 μM	10.9	100
41	Phylligenin	500 μM	25.7	90
42	Protocatechuic acid	500 μM	11.2	60
43	Quebrachitol	250 μM	12.0	100
44	Nodakenin	50 μM	11.9	100
45	Scopolin	250 μM	13.0	100
46	Pectolinarin	250 μM	11.8	100
47	coptisine	50 μM	56.7	90
48	Methyleugerol	250 μM	8.6	100
49	Pulegonel	250 μM	9.4	100
50	Biflorin	500 μM	10.8	90
51	Fluoxetine	1.5 μM	11.0	100
52	Echinocystic acid	50 μM	8.2	100
53	Ursolic acid	25 μM	7.8	100
54	Maslinic acid	50 μM	9.7	100
55	Rubrofusarin	330 μM	11.5	100
56	phylodullcin	330 μM	21.9	70
57	chlorogenic acid	66 μg/ml	6.2	100
58	HBME	0.8 mg/ml	15.5	100
59	swertisin	0.33%	8.7	100
60	swertiamarin	0.33 mg/ml	10.0	100
61	EC-18	0.33 mg/ml	5.8	100

Figure S1.

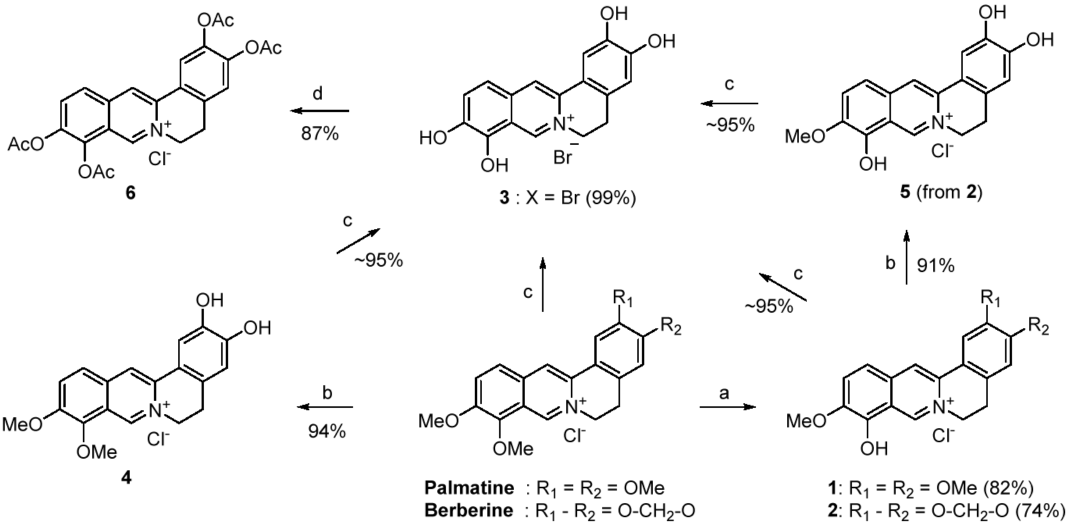

Figure S1. Synthetic scheme of six derivatives from the isoquinolinium core scaffold. Reagents and reaction conditions: (**A**) 190-210 °C, 0.01 atm, 30 min; (**B**) BCl_3, CH_2Cl_2, 0 °C → rt, 6 h; (**C**) BBr_3, CH_2Cl_2, -20 °C --> rt, 6 h; (**D**) Accl, pyridine, 0 °C → rt, 4 h.

Figure S2.

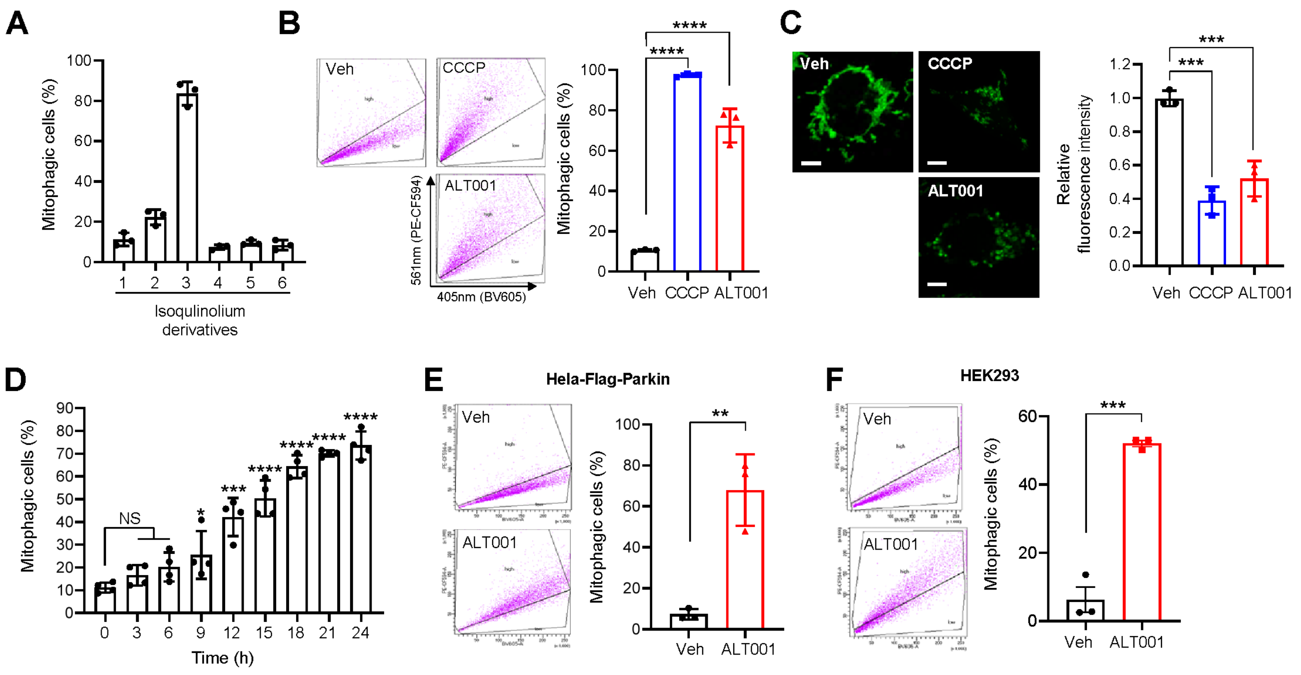

Figure S2. Development and verification of ALT001 (A) BEAS-2B expressing mt-Keima cells were treated with isoqulinolium derivatives (15 μM) for 24 h, and mitophagy levels were analyzed by flow cytometry. The results from three biological replicates are shown as the mean ± SD **(B)** BEAS-2B cells expressing mt-Keima were treated with ALT001 (15 μM) or CCCP (10 μM) for 24 h, and mitophagy levels were analyzed by flow cytometry**.** The results from three biological replicates are shown as the mean ± SD. **(C)** HeLa cells expressing mitoYFP were treated with ALT001 (15 μM) or CCCP (10 μM) for 24 h, and fluorescence intensity was analyzed by confocal microscopy. Scale bar: 10 μm. Quantified fluorescent intensities from three biological replicates with five images per biological repeat are shown on the right as the mean ± SD. (**D)** BEAS-2B cells expressing mt-Keima were treated with ALT001 (15 μM) for the indicated times, and mitophagy levels were analyzed by flow cytometry. The results from four biological replicates are shown as the mean ± SD. (**E-F)** HeLa-Parkin cells (**E**) and HEK293 cells **(F)** expressing mt-Keima were treated with ALT001 (15 μM) for 24 h, and mitophagy levels were analyzed by flow cytometry. The results from three biological replicates are shown as the mean ± SD. Significance was determined by Student's *t test* **(E, F) or** one-way ANOVA **(B, C, D)** with Šidák's multiple-comparison test. *P < 0.05; **P < 0.01; ***P < 0.001; ****P < 0.0001, NS, not significant.

Figure S3.

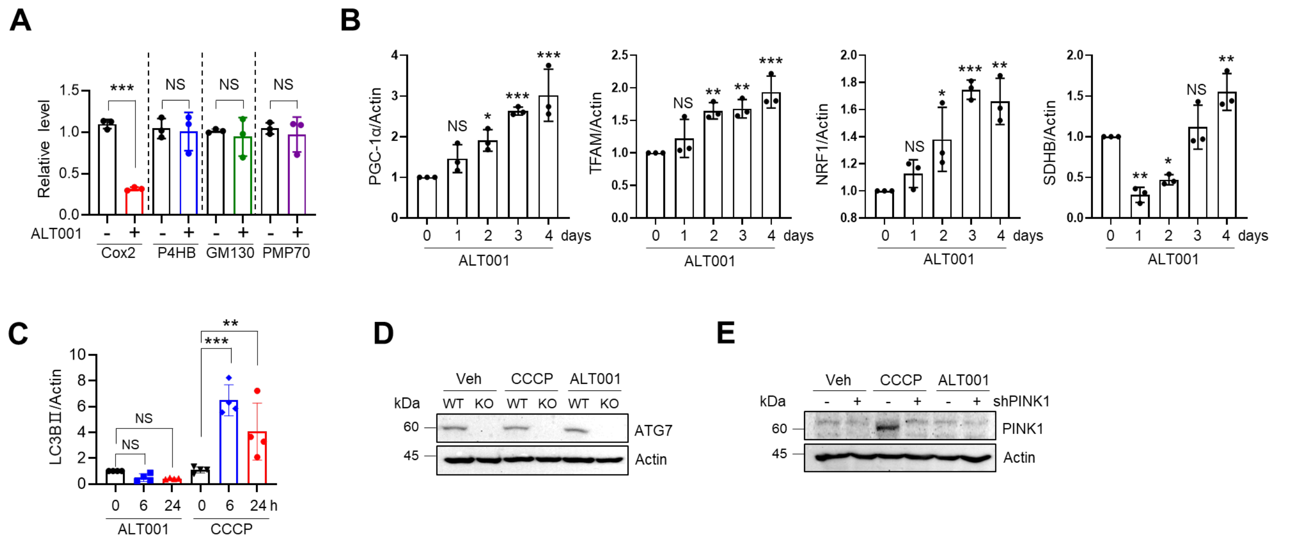

Figure S3. Analysis of mitophagy-specific induction and dependency of the canonical mitophagy pathway of ALT001. (A) Quantitative analysis of the protein levels depicted in Fig. 2D. The data represent the mean ± SD obtained from three independent biological replicates. **(B)** Quantification of the protein levels shown in Fig. 2F. The data represents the mean ± SD obtained from three independent biological replicates. **(C)** Quantification of LC3B type II protein levels shown in Fig. 2G. The data represent the mean ± SD obtained from four independent biological replicates. **(D)** Wild-type (WT) and ATG7 knockout (KO) HeLa cells expressing Parkin were treated with either CCCP (10 μM) for 2 h or ALT001 (15 μM) for 24 h, and western blot analysis was performed using the indicated antibodies. **(E)** BEAS-2B cells expressing control nontargeting shRNA (shPINK1 -) or PINK1 shRNA (shPINK1 +) were treated with either CCCP (10 μM) or ALT001 (15 μM) for 24 h, and western blot analysis was performed using the indicated antibodies. Significance was determined by Student's *t* test **(A)** or one-way ANOVA **(B, C)** with Šidák's multiple-comparison test. *$P < 0.05$; **$P < 0.01$; ***$P < 0.001$.

Figure S4.

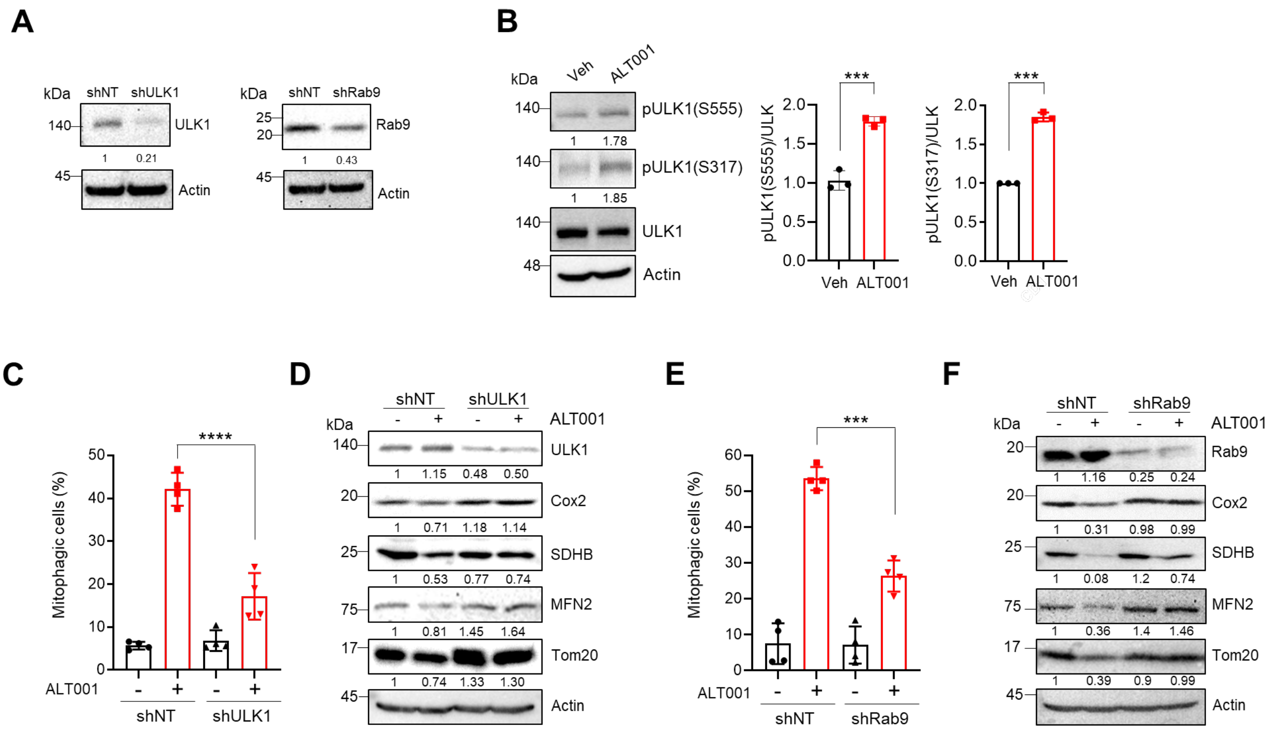

Figure S4. Verification of the alternative mitophagy pathway dependency of ALT001. (A) Protein levels of ULK1 and Rab9 in BEAS-2B cells expressing control nontargeting shRNA (shNT), ULK1 shRNA (shULK1) or Rab9 shRNA (shRab9), as shown in Fig. 3F and G. **(B)** HEK293 cells were treated with ALT001 (15 μM) for 12 h, and western blot analysis was performed using the indicated antibodies. Quantitative analysis of the protein levels shown on the right as the mean ± SD. **(C-F)** HEK293 cells expressing shULK1 **(C-D)** or shRab9 **(E-F)** were treated with ALT001 (15 μM) for 12 h, and mitophagy levels were analyzed by flow cytometry **(C-E)**. Western blot analysis was performed using the indicated antibodies **(D-F)**. The results from four biological replicates **(C-E)** are shown as the mean ± SD. Numbers below the corresponding western blot represent densitometric analysis normalized to Actin. Significance was determined by Student's *t* test **(B)** or two-way ANOVA **(C-E)** with Šidák's multiple-comparison test. ****$P < 0.0001$.

Figure S5.

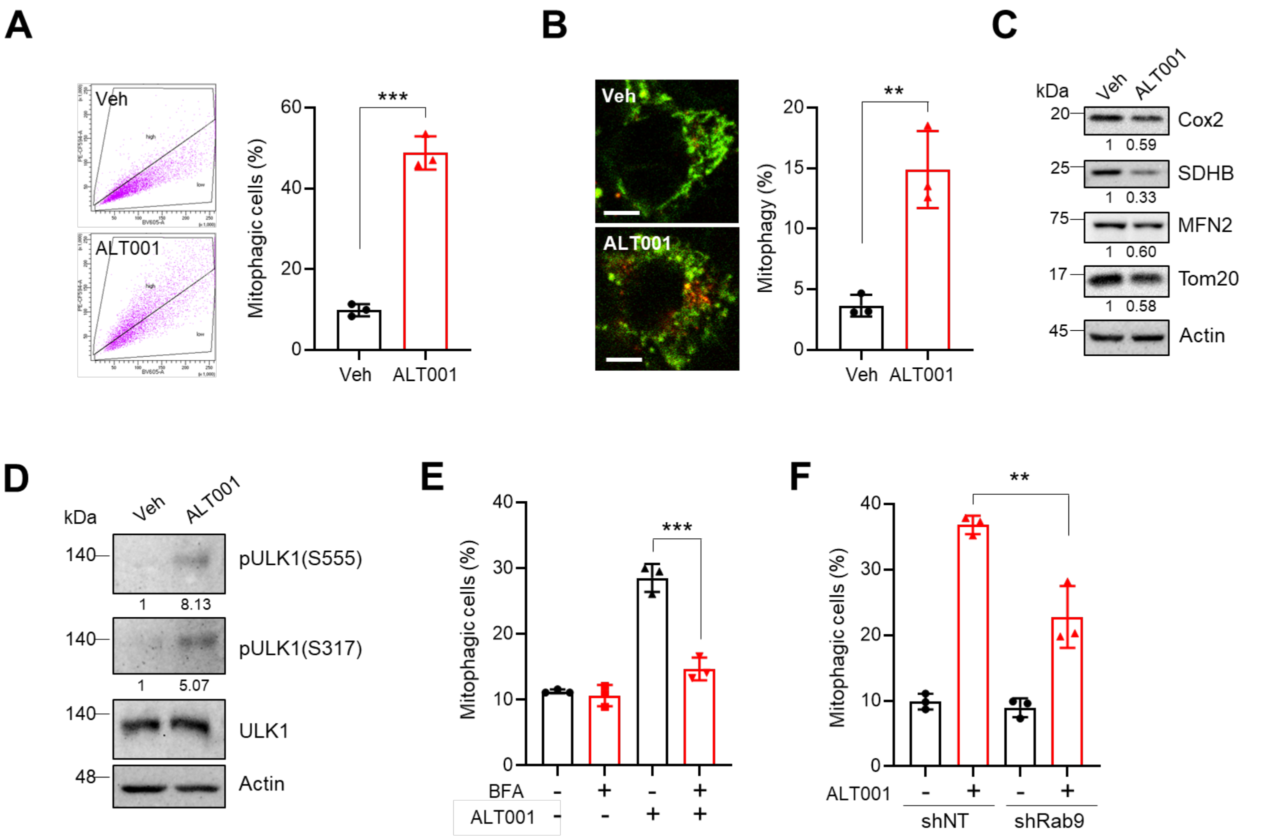

Figure S5. Verification of mitophagy induction by ALT001 in SH-SY5Y cells. (A-C) SH-SY5Y cells expressing mt-Keima were treated with ALT001 (15 μM) for 24 h, and mitophagy levels were analyzed by flow cytometry **(A)** and confocal microscopy **(B)**. The results from three biological replicates are shown on the right as the mean ± SD. Western blot analysis was performed using the indicated antibodies **(C)**. Numbers below the corresponding blot represent densitometric analysis normalized to actin. **(D)** SH-SH5Y cells were treated with ALT001 (15 μM) for 12 h, and western blot analysis was performed using the indicated antibodies. Numbers below the corresponding western blot represent densitometric analysis normalized to actin. **(E)** SH-SY5Y cells expressing mt-Keima were treated with ALT001 (15 μM) together with brefeldin A (BFA; 0.01 μg/ml) for 12 h, and mitophagy levels were analyzed by flow cytometry. The results from three biological replicates are shown as the mean ± SD. **(F)** SH-SY5Y cells expressing control nontargeting shRNA (shNT) or Rab9 shRNA (shRab9) were treated with ALT001 (15 μM) for 12 h, and mitophagy levels were analyzed by flow cytometry. The results from three biological replicates are shown as the mean ± SD. Significance was determined by Student's *t test* **(A, B)** or two-way ANOVA **(E, F)** with Šidák's multiple-comparison test. $**P < 0.01$; $***P < 0.001$; NS, not significant.

Figure S6.

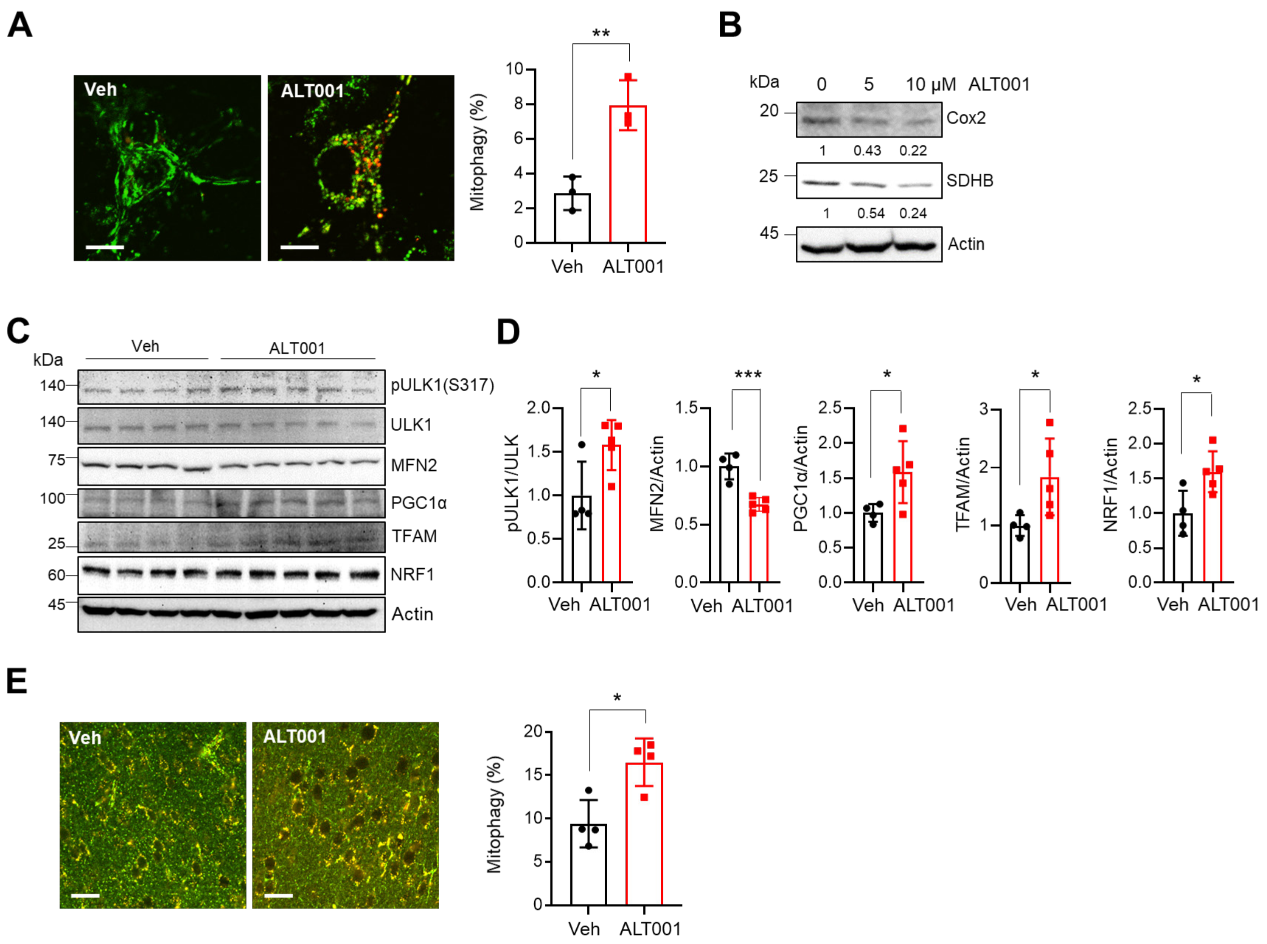

Figure S6. Induction of mitophagy *in vivo* by ALT001. (A) Primary cortical neurons were isolated from FVB-mt-Keima mice and treated with ALT001 (5 μM) for 8 h. The levels of mitophagy were determined by confocal microscopy. Scale bar: 10 μm. Quantified mitophagy levels from three biological replicates with five images per biological repeat are shown on the right as the mean ± SD. **(B)** Primary cortical neurons were isolated from mt-Keima mice and treated with ALT001 for 24 h at the indicated concentration. Western blotting analysis was performed using the indicated antibodies. Numbers below the corresponding blot represent densitometric analysis normalized to Actin. **(C-D)** mt-Keima male mice were treated with ALT001 (1 mg/kg) or vehicle (Veh) for 7 days via nasal administration. Hippocampal tissue was isolated and western blot analysis was performed using the indicated antibodies **(B)**. Quantified protein levels are shown as the mean ± SD **(C)**. (n = 4 or 5 per group) **(E)** FVB-mt-Keima male mice were treated with ALT001 (1 mg/kg) by intranasal administration daily for 7 days, and mt-Keima fluorescence was analyzed by confocal microscopy. Scale bar: 20 μm. Quantified mitophagy levels from four mice with two images per mouse are shown on the right as the mean ± SD. Significance was determined by Student's *t test* **(A, D, E)** **P* < 0.05;

**$P < 0.01$; ***$P < 0.001$.

Figure S7.

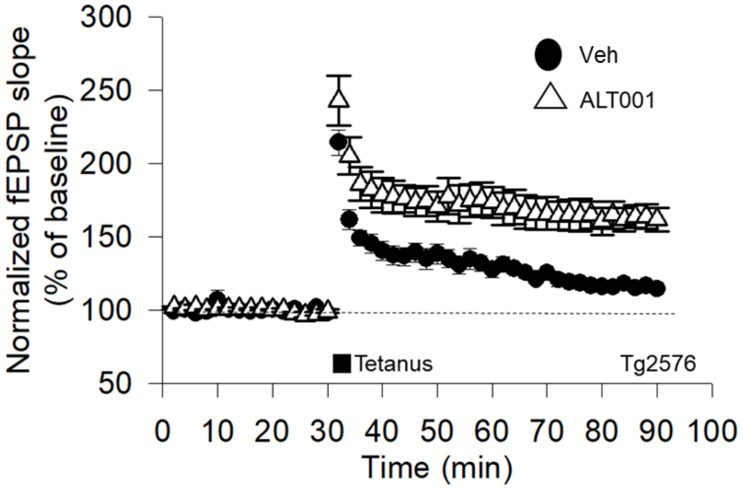

Figure S7. ALT001 restores memory function in the Tg2576 mouse model. Hippocampal LTP was assessed in 9-month-old Tg2576 male mice treated with ALT001 (1 mg/kg) (triangle, n = 6) or vehicle (Veh, n = 5) by intranasal administration daily for 4 weeks. Error bars indicate the SEM.

Figure S8.

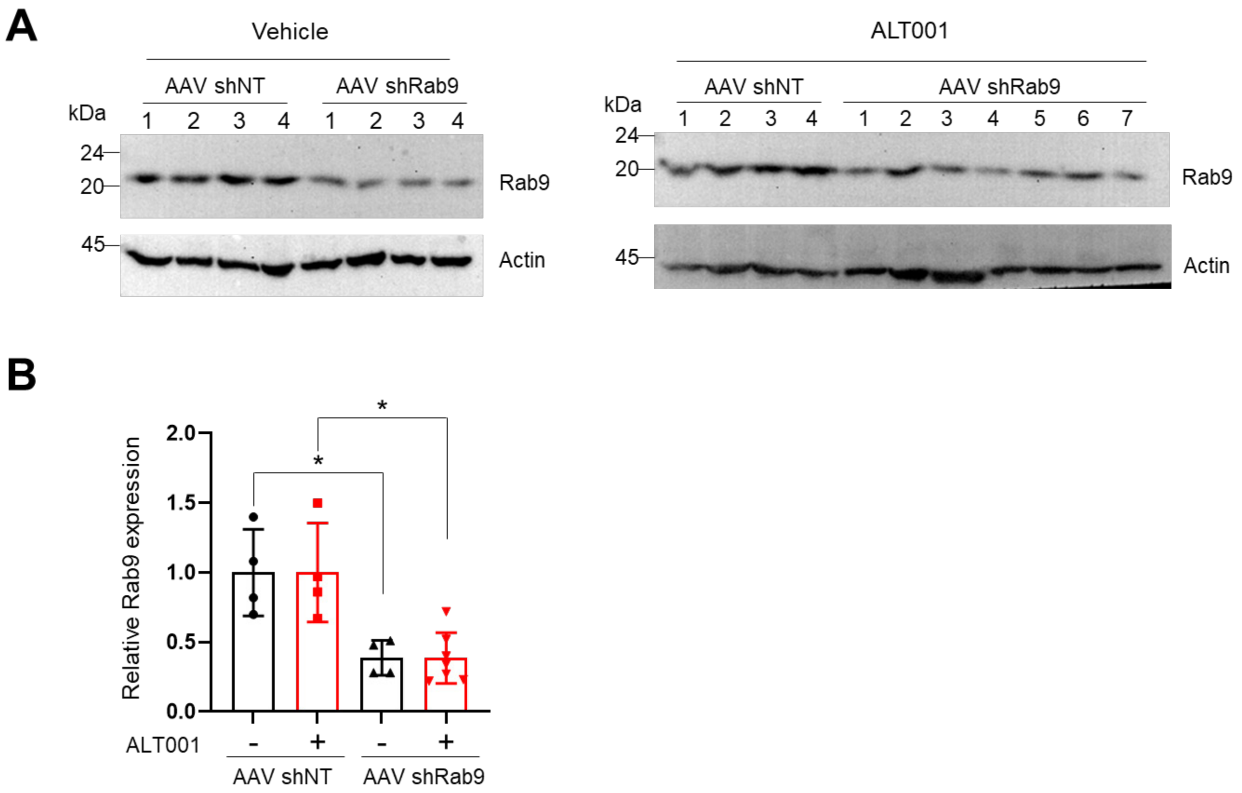

Figure S8. Verification of Rab9 knockdown upon Rab9 shRNA AAV injection into hippocampus. (**A**) FVB-mt-Keima mice were injected with Rab9 shRNA AAV (AAV shRab9) or control shRNA AAV (AAV shNT) into hippocampus. After 2 weeks injection, vehicle (Veh) or ALT001 (1 mg/kg) treated via nasal administration for 7 days, the hippocampal tissue was isolated and western blot analysis was performed using the indicated antibodies (AAV shNT vehicle, n = 4; AAV shNT ALT001, n = 4; AAV shRab9 shNT, n = 4, AAV shRab9 ALT001 n = 7). (**B**) Quantified protein levels shown in (**A**) are presented as the mean ±SD. Significance was determined by two-way ANOVA with Šidák's multiple-comparison test. *$P < 0.05$.
